# Health impact of self-help groups scaled-up statewide in Bihar, India

**DOI:** 10.7189/jogh.10.021006

**Published:** 2020-12

**Authors:** Kala M Mehta, Laili Irani, Indrajit Chaudhuri, Tanmay Mahapatra, Janine Schooley, Sridhar Srikantiah, Safa Abdalla, Victoria C Ward, Suzan L Carmichael, Jason Bentley, Andreea Creanga, Jess Wilhelm, Usha Kiran Tarigopula, Debarshi Bhattacharya, Yamini Atmavilas, Priya Nanda, Yingjie Weng, Kevin T Pepper, Gary L Darmstadt, for the Ananya Study Group, Yamini Atmavilas, Debarshi Bhattacharya, Jason Bentley, Evan Borkum, Suzan Carmichael, Indrajit Chaudhuri, Andreea Creanga, Gary L. Darmstadt, Priyanka Dutt, Laili Irani, Tanmay Mahapatra, Kala M. Mehta, Radharani Mitra, Wolfgang A. Munar, Priya Nanda, Kevin T. Pepper, Hina Raheel, Anu Rangarajan, Niranjan Saggurti, Padmapriya Sastry, Hemant Shah, Sridhar Srikantiah, Victoria Ward, Yingjie Weng, Dilys Walker, Jess Wilhelm

**Affiliations:** 1 Department of Pediatrics, Stanford University School of Medicine, Stanford, California, USA; 2 Department of Epidemiology and Biostatistics, University of California San Francisco, San Francisco, California, USA; 3 Population Council, New Delhi, India; 4 Project Concern International, Delhi, India and San Diego, California, USA; 5 CARE India, Patna, India; 6 Center for Population Health Sciences, Stanford University School of Medicine, Palo Alto, California, USA; 7 Quantitative Sciences Unit, Department of Medicine, Stanford University School of Medicine, Stanford, California, USA; 8 Department of International Health, Johns Hopkins Bloomberg School of Public Health, Baltimore, Maryland, USA; 9 Bill and Melinda Gates Foundation, Delhi, India

## Abstract

**Background:**

The objective of this study was to assess the impact of self-help groups (SHGs) and subsequent scale-up on reproductive, maternal, newborn, child health, and nutrition (RMNCHN) and sanitation outcomes among marginalised women in Bihar, India from 2014-2017.

**Methods:**

We examined RMNCHN and sanitation behaviors in women who were members of any SHGs compared to non-members, without differentiating between types of SHGs. We analysed annual surveys across 38 districts of Bihar covering 62 690 women who had a live birth in the past 12 months. All analyses utilised data from Community-based Household Surveys (CHS) rounds 6-9 collected in 2014-2017 by CARE India as part of the Bihar Technical Support Program funded by the Bill & Melinda Gates Foundation. We examined 66 RMNCHN and sanitation indicators using survey logistic regression; the comparison group in all cases was age-comparable women from the geographic contexts of the SHG members but who did not belong to SHGs. We also examined links between discussion topics in SHGs and changes in relevant behaviours, and stratification of effects by parity and mother’s age.

**Results:**

SHG members had higher odds compared to non-SHG members for 60% of antenatal care indicators, 22% of delivery indicators, 70% of postnatal care indicators, 50% of nutrition indicators, 100% of family planning and sanitation indicators and no immunisation indicators measured. According to delivery platform, most FLW performance indicators (80%) had increased odds, followed by maternal behaviours (57%) and facility care and outreach service delivery (22%) compared to non-SHG members. Self-report of discussions within SHGs on specific topics was associated with increased related maternal behaviours. Younger SHG members (<25 years) had attenuated health indicators compared to older group members (≥25 years), and women with more children had more positive indicators compared to women with fewer children.

**Conclusions:**

SHG membership was associated with improved RMNCHN and sanitation indicators at scale in Bihar, India. Further work is needed to understand the specific impacts of health layering upon SHGs. Working through SHGs is a promising vehicle for improving primary health care.

**Study registration:**

ClinicalTrials.gov number NCT02726230.

Addressing poverty, hunger, health, and gender equality are key Sustainable Development Goals but few interventions have strong evidence for improving outcomes across financial, social and health sectors simultaneously [1]. Self-help groups (SHGs) have an expanding evidence base of influence on income, asset ownership, savings, and household ability to withstand economic shocks [2]. Evidence is growing for non-financial outcomes as well, including political empowerment, social cohesion, collective action and personal agency, and health [3].

SHGs seek to improve the lives of their members using a group/collective approach over a purely individual one. In the West, these groups are known primarily for providing mental health peer support, often led by a facilitator, as in the case of Alcoholics Anonymous [4]. In the global health context and that of India, these groups primarily address the financial and social needs of poor and maginalised populations. Although they were founded as a vehicle to increase economic empowerment using microcredit, they have grown to address non-financial social issues primarily for rural women and increasingly through collective action strategies [5]. They are also an important mechanism used by the Indian Government to engage poor women in central systems, including health and poverty reduction schemes [5]. At the household and individual level, SHG participation can impact a woman’s ability to manage her own financial circumstances and potentially shift household gender-based decision-making to become more equitable, ultimately benefitting herself and her family [6].

The theoretical frameworks that underlie SHG implementation and evaluation derive primarily from a participant empowerment model, wherein group members become empowered as individuals as well as engage in collective action, increasing self-efficacy and participation in specific dialogues about their circumstances, thereby increasing women’s agency and autonomy, including for health-related decision-making [7]. Under these frameworks, the most frequently studied outcomes of SHGs are financial, ranging from increased savings, access to credit and microenterprise, income for small enterprises, and ownership of assets [3,8]. In terms of health, two systematic reviews cite strong evidence that SHG membership is associated with reductions in maternal mortality [odds ratio (OR)  = 0.63, 95% confidence interval (CI)  = 0.32-0.94] and neonatal mortality (OR = 0.77, 95% CI = 0.65-0.90) [8-10]. Similarly, Seward et al showed roughly a 2-fold increase in health-promoting behaviours during and after home deliveries among SHG members engaged in health education and participatory learning and action activities [11]. Connection to a frontline worker (FLW) such as an Accredited Health Social Activist (ASHA) also appears to be an important contributor to health impact [12]. However, not all trials show an SHG advantage [13], and data are lacking on the impact of SHGs when scaled. In India, SHGs started over 30 years ago with a direct tie to microfinance. SHGs are scaling at a rapid pace throughout India and include an estimated 200 million members in 8.7 million SHGs, most with the goal of economic empowerment but with increased interest in applications to health promotion [14].

Our study examines SHG membership in a time period of rapid change and expansion in SHGs in Bihar (2014-2017). We examine the health impact of SHGs at scale, but do not differentiate the type of SHG; this is the subject of a subsequent manuscript [15]. The current study extends prior findings to examine indicators across the RMNCHN continuum of care at scale, including characteristics associated with SHG performance [16].

## METHODS

### Context for SHGs in Bihar

The *Ananya* initiative (2010-2013) and subsequent Bihar Technical Support Program (BTSP) (2014-present) had the primary goal of strengthening the capacity of the Government of Bihar (GoB) to improve reproductive, maternal, newborn, child health and nutrition (RMNCHN) outcomes statewide. Several NGOs including CARE India, BBC Media Action, and Project Concern International (PCI) launched or strengthened a range of RMNCHN interventions during the pilot period of 2012 through 2013 in eight focus districts: Patna, Saharsa, East Champaran, West Champaran, Samastipur, Bengusarai, Gopalganj and Khagaria [17-22. Beginning in 2014 to present, interventions were scaled up by the GoB statewide across all 38 districts with techno-managerial support from CARE (through the BTSP), PCI − through the JEEVIKA Technical Support Program (JTSP), and others [21,23]. Though there were several nuances in SHG funding sources, formation and types, we do not address these in the current manuscript. Most notably, SHG health-layered interventions are described and examined in a subsequent paper (Figure S1 in the **Online Supplementary Document**).

### Self-help groups

Over the course of this study, from 2014-2017, SHGs in Bihar were scaled-up statewide to all 38 districts [24]. For the current analysis, we used respondent-identification of SHG membership. We did not distinguish whether the SHG was government- or non-governmental organization (NGO)-led, or who funded the SHG. SHGs are variable in their structure and function but generally are organised around three main themes. Savings groups are typically formed by women but can be promoted by either NGOs or the government; these usually have 10-20 members [8,25. Women’s health groups, a type of SHG, typically have a leader who provides training on specific health topics, have regularly scheduled meetings with 10-20 members and have an express mission to increase women’s empowerment/demand for access to health-related resources. Agriculture-related groups typically are comprised of both women and men (typically 12-30 members) and aim to increase connections to markets as well as create risk-pooling [8]. In qualitative studies, women belonging to these groups report that group membership is positive and decreases poverty [26]. In Bihar, groups generally were organised around access to microfinance and some, but not all, had specific embedded health objectives or health layering [16].

### Evaluation

#### Community-based Household Surveys

The data for this study came from four rounds of Community-based Household Surveys (CHS) collected in 2014-2017 by CARE India to monitor progress with implementation of the BTSP funded by the Bill & Melinda Gates Foundation. These repeated cross-sectional surveys of women used a Lot Quality Assurance Plus Sampling (LQAS)-like methodology [27]. The methodology of survey administration and calculation of survey weights has been described previously [21]. In brief, sampling was conducted in all 38 districts, including each block within each district. For each block, the sample size was proportionate to the known population of the block, subject to a minimum of 19 households. Within blocks, Anganwadi Centres (AWCs) served as primary sampling units. AWCs were randomly selected. Within AWCs, a starting household was identified by random selection from the household roster of the AWC. Survey enumerators visited the fifth household from the index household, using a right-hand rule, to assess eligibility. Enumerators visited the next fifth household until one mother of a child in each of five age-groups (0-2, 3-5, 6-8, 9-11 and 12-23 months) was surveyed. Stratification by age-group (0-2, 3-5, 6-8, 9-11 and 12-23 months) was used to limit recall bias and enable assessment of key domains that were the focus of program implementation across the continuum of care for health, nutrition and sanitation. This provided a sample size across all districts of the state of 15 687 for each of the five age groups. Data collection teams from CARE India’s Concurrent Measurement and Learning Unit, which was independent of implementation, went to each randomly selected household and administered surveys specific for mothers of babies in each of the five age groups to enable focus on age-specific indicators. Data quality control included mandated spot checks and back-checks by an independent team of data supervisors for 15% of data collected.

We utilised CHS survey questions in rounds 6-9 about participation in SHGs to identify women who self-reported as members of an SHG compared to age-comparable women who reported that they were not SHG members. In CHS rounds 7-8, respondents were asked questions about specific topics which were discussed during SHG meetings, and in CHS rounds 7-9, members of SHGs were asked how long they had participated in a group. We also identified survey questions in CHS about characteristics of women associated with SHG membership including age and parity.

#### Indicator selection and categorisation

We focused initially on 66 indicators which broadly reflected the continuum of care from conception through infancy (Table S1 in the **Online Supplementary Document**). We restricted our analytical cohort to women with children aged 0-2 months for antenatal, delivery and postnatal care and postpartum family planning indicators; 9-11 months for immunisation, nutrition and family planning indicators, and 12-23 months for sanitation and family planning indicators. These indicators were selected because they were most relevant to the health modules layered on SHGs while still representing a broad array of indicators in the continuum of care.

Prior to analysis, indicators were grouped into the following domains according to the continuum of care: antenatal care (ANC) and birth preparedness, delivery (childbirth care), postnatal care, nutrition, immunisation, family planning, and sanitation (Table S1 in the **Online Supplementary Document**). For each continuum-of-care domain, we then classified the indicators into one of three delivery platforms: facility care and outreach service delivery, FLW performance and behaviour, and mother’s behaviour.

### Statistical analysis

We first compared women who were members of a SHG to non-SHG members in rounds 6-9 of the CHS in all 38 districts statewide (2014-2017). We calculated the odd ratios (ORs) and 95% confidence intervals (Cis) for all 66 indicators using survey logistic regression, comparing SHG members to non-members using an indicator term. The *P*-values in all models were adjusted for multiple comparisons using the False Discovery Rate-controlling method of Benjamini-Hochberg [28] and were additionally adjusted for maternal age and focal child gender. Models incorporated study design using sampling weights. For all models we also performed a sensitivity adjustment for additional variables including household size, number of children, asset index, Hindu religion, Scheduled Tribe/Scheduled Caste, literacy level, whether the family lived with nuclear or extended family members, both mother’s and father’s educational level and whether the family lives in a *pucca* (ie, solid, permanent, built of substantial material such as stone, brick, cement, concrete, or timber) house. As most of these variables were highly collinear with the primary predictor, SHG membership, we present data based on adjustment for maternal age and focal child gender for our primary analyses.

We examined the prevalence of discussions of various topics in SHGs and whether the discussions were associated with relevant indicators, comparing women in SHGs who had the topic discussed compared to women in SHGs who did not. Discussion topics were only queried in CHS survey rounds 7 and 8, not in rounds 6 or 9. Thus, for example, for rounds 7 and 8 we examined whether a discussion on contraception was associated with increased family planning behaviour for the respondent. Discussions encompassed: 1) birth preparedness, 2) newborn care, 3) breastfeeding and complementary feeding, and 4) credit and savings. We examined whether time spent in SHGs, measured in months, impacted whether a woman saved money for delivery or received advice about whether to do so. Given the differing needs of younger compared to older SHG members, and that SHG membership and activities are typically formulated for reproductive age women engaged in income generation and/or livelihoods, we undertook a stratified analysis of SHG members in CHS rounds 7-9 who were above and below the median age of 25 years. In addition, we similarly stratified by parity, contrasting pregnant women/women with one child, two children, or 3 or more children. Further, we examined touchpoints with the primary health care system as a potential moderator of positive associations of SHG membership with RMNCHN indicators. We calculated a point score for each interaction a maternal respondent had with a FLW (either an ASHA, AWW, or ANM), that is, a positive response to any of five questions about FLW/beneficiary interactions: FLW visit in the last trimester, FLW present at birth, FLW visit in the first 24 hours after childbirth, FLW visit in the first week after childbirth, and FLW visit in the first month after childbirth. We examined the impact of FLW touchpoints on indicators, stratified by SHG membership.

### Ethical considerations

Permission for access and terms of CHS data use were agreed with CARE India through a data sharing agreement and approved by the Stanford University Institutional Review Board protocol #39719. This study is part of the *Ananya* program which was registered with ClinicalTrials.gov number NCT02726230.

## RESULTS

### Study population

Socioeconomic and demographic characteristics of the CHS study population for rounds 6-9 during 2014-2017 are displayed in **Table 1**. Overall, compared to non-members, SHG members were slightly older, had more children, were more often from a Scheduled Caste and Hindu, were less likely to have formal education/literacy, and had poorer living conditions (ie, less likely to dwell in a *pucca* house) compared to non-members studied. Thus, consistent with the intent of SHGs to reach marginalised women, SHG member women were, in general, at greater social disadvantage than non-SHG members, predisposing them to worse health indicators.

**Table 1:** Characteristics of maternal household respondents in the Community-based Household Surveys according to Self-help group (SHG) membership, rounds 6-9 (2014-2017) in Bihar, India*

	Not SHG members (84.1%)		SHG members (15.9%)	
	**Frequency**	**Row percent or mean**	**Frequency**	**Row percent or mean**
**Woman’s age in years§**	52 755	24.0	9935	25.4
**Household size**	52 755	7.8	9935	7.3
**Average number of children**	52 755	2.6	9935	3.3
**Average number of adults in household**	52 755	5.2	9935	4.0
**Highest educational level for woman†**	5 755	4.2	9935	2.8
**Highest educational level for husband†**	50 961	5.6	9572	4.3
**Gender of child:‡**				
Female	25 240	47.8	4799	48.2
Male	27 515	52.2	5136	51.8
**Nuclear family living in household only:**†				
No	35 074	66.4	4959	49.4
Yes	17 681	33.6	4976	50.6
**Literacy level of the husband:**†				
No	28 335	53.0	6388	64.0
Yes	24 420	47.0	3547	36.0
**Literacy level of the woman:**†				
No	20 210	37.8	4603	46.4
Yes	32 473	62.2	5316	53.6
**Caste:**†				
General/Other	6519	12.5	550	5.2
Other Backward Caste	32 861	62.2	5621	56.2
Scheduled Caste	12 360	23.4	3568	36.6
Scheduled Tribe	1015	1.9	196	2.0
**Religion:**†				
Buddhist	2	0.0	0	.
Christian	53	0.1	5	0.1
Hindu	44 709	83.9	8848	88.7
Jain	4	0.0	1	0.0
Muslim	7972	16.0	1080	11.2
Other	9	0.0	1	0.0
Sikh	1	0.0	0	.
**Hindu religion and Scheduled Caste/Scheduled Tribe (SC/ST):**†				
Hindu, non-SC/ST	31 604	59.1	5124	50.5
Hindu, SC/ST	13 105	24.7	3724	38.2
Non-Hindu	8041	16.1	1087	11.3
**Dwelling structure:**†				
*Kucha*	16 212	30.3	3366	34.6
*Pucca*	9403	17.2	972	9.4
Semi-*pucca*	27 140	52.4	5597	56.0

*Focal and non-focal districts statistically were similar in terms of demographics, reported by SHG membership

†Items indicate statistically significant difference between SHG and non-SHG members using complex survey based t-tests (surveyttest) and chi-squared tests (survey freq) where appropriate.

‡In this table, we present data from the 0-2 months of age of focal child data sources.

§Inclusion criteria were that a maternal household respondent had a child <12 months ago.

### Community-based Household Survey results

#### Continuum-of-care domains

**Figure 1:**
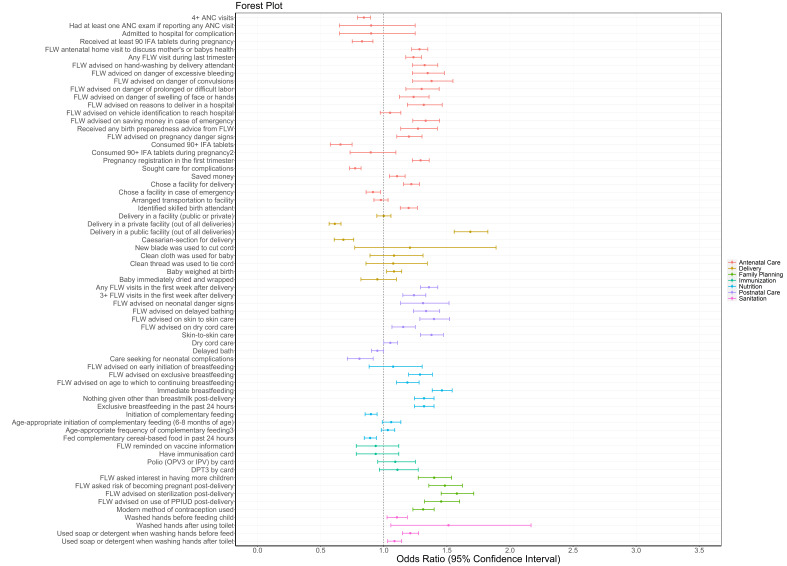
Forest plot of odds ratios and 95% confidence intervals of health, nutrition and sanitation indicators by continuum-of-care domain for self-help group members compared to non-members as measured by the Community-based Household Surveys rounds 6-9 during scale-up (2014-2017) statewide in Bihar, India. All models presented were adjusted for age of the mother and sex of the focal child. These models also accounted for the study’s complex design by applying study weights. 1 – Out of those who received 90+ IFA during pregnancy. 2 – 3+ times for 9-11 month-old children. ANC – antenatal care, DPT – diphtheria-pertussis-tetanus, FLW – frontline worker, IFA – iron-folic acid, IPV – inactivated polio vaccine, OPV – oral polio vaccine, PPIUD – postpartum intrauterine device.

Odds for indicators showing significantly higher levels ranged from near unity (OR = 1.10, 95% CI = 1.02-1.19) for washed hands before feeding the child to nearly 70% increased (OR = 1.69, 95% CI = 1.56-1.82) for delivery in a public facility. The majority of antenatal indicators (60%) had significantly higher odds for SHG members compared to non-members in rounds 6-9 during the scale-up period (**Figure 1**, Table S2 in the **Online Supplementary Document**). A small minority of antenatal care indicators (5%) were significantly lower for SHG members, including 4+ ANC visits and receipt and consumption of 90 iron-folic acid (IFA) tablets. Among delivery indicators, about one fifth showed higher odds for SHG members compared to non-members. For more than three-quarters (78%) of postnatal care indicators, odds were significantly higher for SHG members compared to non-members in rounds 6-9, ranging from 1.15 (95% CI = 1.07-1.25) for frontline worker (FLW) advising dry cord care to 1.40 (95% CI = 1.29-1.52) for maternal behaviour of skin-to-skin care.

Of the nutrition indicators, 50% had significantly higher odds for SHG members. There was a significant increase in odds of immediate breastfeeding (OR = 1.46, 95% CI = 1.39-1.55) but the odds of complementary feeding initiation decreased (OR = 0.90, 95% CI = 0.85-0.95). No immunisation indicators increased. All family planning indicators were significantly higher, ranging from OR = 1.31 (95% CI = 1.23-1.40) for use of modern methods of family planning to OR = 1.56 (95% CI = 1.44-1.64) for FLW advice on adoption of sterilisation. All sanitation indicators showed significantly higher odds for SHG members compared to non-members, with the highest OR for washing hands after using the toilet (OR = 1.51, 95%CI = 1.06-2.17).

#### Delivery platforms

**Figure 2:**
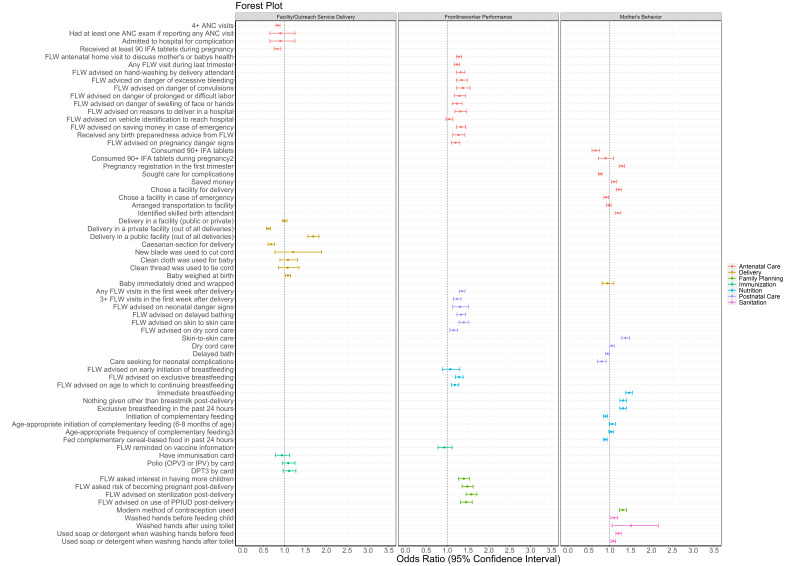
Forest plot of odds ratios and 95% confidence intervals of health, nutrition and sanitation indicators for self-help group members compared to non-members by delivery platform as measured by the Community-based Household Surveys rounds 6-9 during scale-up (2014-2017) statewide in Bihar, India. All models presented were adjusted for age of the mother and sex of the focal child. These models also accounted for the study’s complex design by applying study weights. 2 – Out of those who received 90+ IFA during pregnancy. 3 – 3+ times for 9-11 month-old children. ANC – antenatal care, DPT – diphtheria-pertussis-tetanus, FLW – frontline worker, IFA – iron-folic acid, IPV – inactivated polio vaccine, OPV – oral polio vaccine, PPIUD – postpartum intrauterine device.

When evaluated by delivery platform, a large majority of FLW performance indicators (80%) had increased odds in SHG members compared to non-members (**Figure 2**, Table S2 in the **Online Supplementary Document**). Maternal behaviours also had increased odds for the majority of indicators (57%). For indicators of facility care and outreach service delivery, 22% were associated with an increased OR  in SHG members.

#### Discussion topics

Using data from CHS rounds 7 and 8 about the discussions which took place in SHGs, we calculated the odds of behaviours/actions according to whether the maternal respondent reported specific types of discussions during the SHG meetings. A report of discussing birth preparedness in the SHG was associated with four times the odds (OR = 4.1, 95% CI = 1.2-14.4) of having had any antenatal visit by a FLW, compared to women in SHGs who did not report that birth preparedness was discussed. Maternal respondents who reported discussing newborn care in the SHG had almost twice the odds of having engaged in newborn skin-to-skin care (OR = 1.85, 95% CI = 1.48-2.34). Women who reported discussing breastfeeding topics in SHGs had over 40% higher odds of having engaged in breastfeeding in the first hour after birth (OR = 1.42, 95% CI = 1.13-1.77). Maternal respondents who reported discussing credit and savings during SHG meetings were 20% more likely to save money for delivery (OR = 1.2, 95% CI = 1.04-1.38).

#### Time spent in SHGs

**Figure 3:**
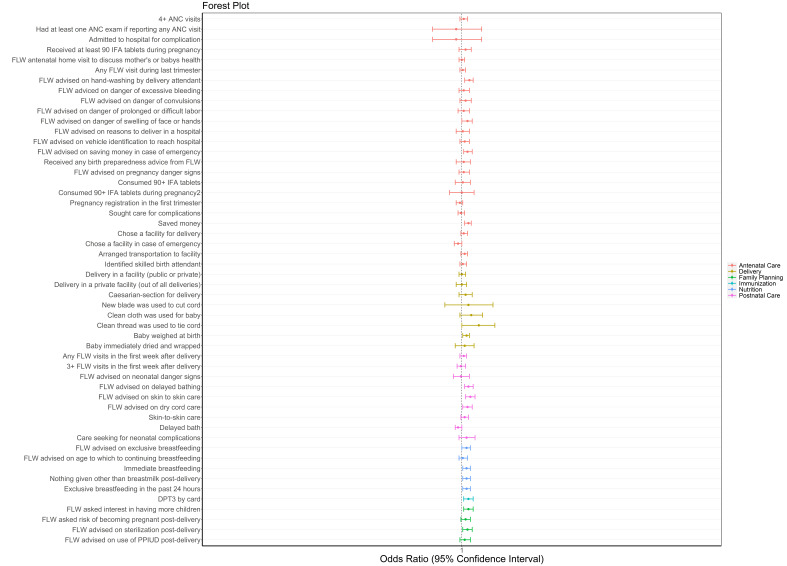
Forest plot of odds ratios and 95% confidence intervals of health, nutrition and sanitation indicators for increased time spent in months in self-help groups as measured by rounds 6-9 of the Community-based Household Surveys during scale-up (2014-2017) statewide in Bihar, India. All models presented were adjusted for age of the mother and sex of the focal child. These models also accounted for the study’s complex design by applying study weights. 2 – Out of those who received 90+ IFA during pregnancy. ANC – antenatal care, DPT – diphtheria-pertussis-tetanus, FLW – frontline worker, IFA – iron-folic acid, IPV – inactivated polio vaccine, PPIUD – postpartum intrauterine device.

We also examined the role of time spent in a SHG. For each increased month in a SHG, a woman had small increased likelihood of a few improved behaviors (**Figure 3**, Table S3 in the **Online Supplementary Document**). They had 0.7% higher odds of saving money for delivery per month (OR = 1.007, 95% CI = 1.001-1.010), 0.5% higher odds of breastfeeding immediately after delivery (OR = 1.005, 95% CI = 1.001-1.009) and 0.5% higher odds of exclusively breastfeeding for the past 24 hours (OR = 1.005, 95% CI = 1.001-1.009).

#### Characteristics of women in SHGs

**Figure 4:**
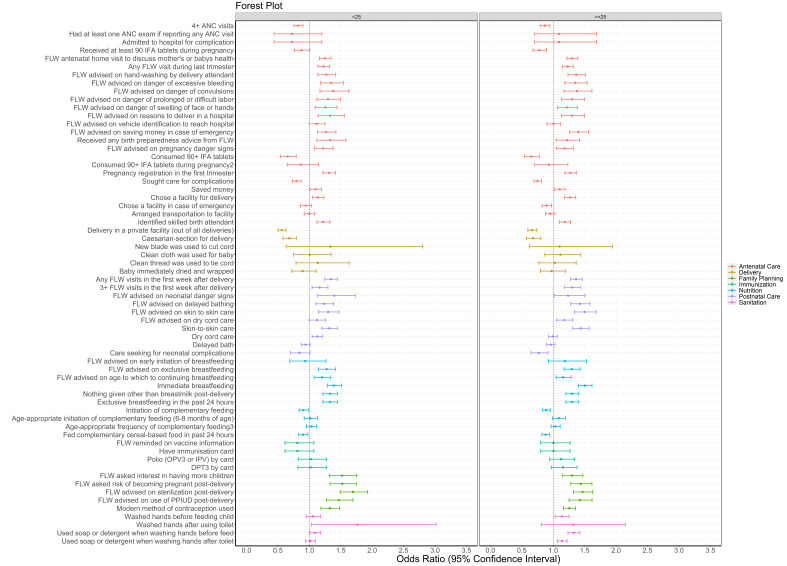
Forest plot of odds ratios and 95% confidence intervals of health, nutrition and sanitation indicators for self-help group members sub-categorised into those who were younger (<25 years old, 1) or older (≥25 years old, 0) compared to age-comparable non-members as measured by Community-based Household Surveys rounds 6-9 during scale-up (2014-2017) statewide in Bihar, India. All models presented were adjusted for age of the mother and sex of the focal child. These models also accounted for the study’s complex design by applying study weights. 2 – Out of those who received 90+ IFA during pregnancy. 3 – 3+ times for 9-11 month-old children. ANC – antenatal care, DPT – diphtheria-pertussis-tetanus, FLW – frontline worker, IFA – iron-folic acid, IPV – inactivated polio vaccine, OPV – oral polio vaccine, PPIUD – postpartum intrauterine device.

**Figure 5:**
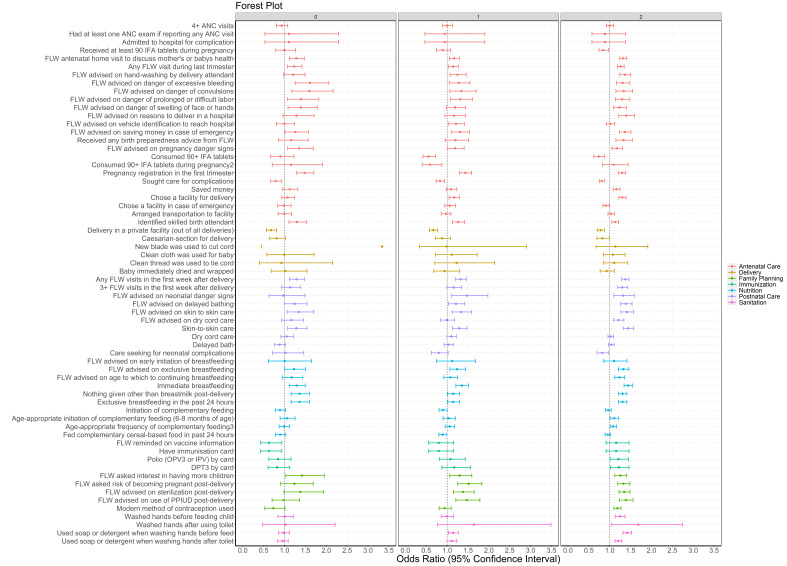
Forest plot of odds ratios and 95% confidence intervals of health, nutrition and sanitation indicators for self-help group members with 0-1 child, 2 children, or 3 or more children compared to parity-comparable non-SHG-members as measured by Community-based Household Surveys rounds 6-9 during scale-up (2014-2017) statewide in Bihar, India. All models presented were adjusted for age of the mother and sex of the focal child. These models also accounted for the study’s complex design by applying study weights. 2 – Out of those who received 90+ IFA during pregnancy. 3 – 3+ times for 9-11 month-old children. ANC – antenatal care, DPT – diphtheria-pertussis-tetanus, FLW – frontline worker, IFA – iron-folic acid, IPV – inactivated polio vaccine, OPV – oral polio vaccine, PPIUD – postpartum intrauterine device.

ORs for indicators associated with SHG membership were generally lower in younger women <25 years of age compared to older women (**Figure 4**, Table S4 in the **Online Supplementary Document**). Of the 64 indicators tested, 48 (55%) had increased odds for older compared to younger SHG members; the remainder were comparable in the two age groups, and three models were not calculable. Increased odds were seen for 63% of RMNCHN + sanitation indicators for women with 3 or more children, compared to 42% for women with 2 children and 29% for women with 1 child or who were pregnant; for 5 models it was not possible to calculate ORs due to insufficient power (**Figure 5**, Table S5 in the **Online Supplementary Document**).

#### FLW-beneficiary interactions

**Figure 6:**
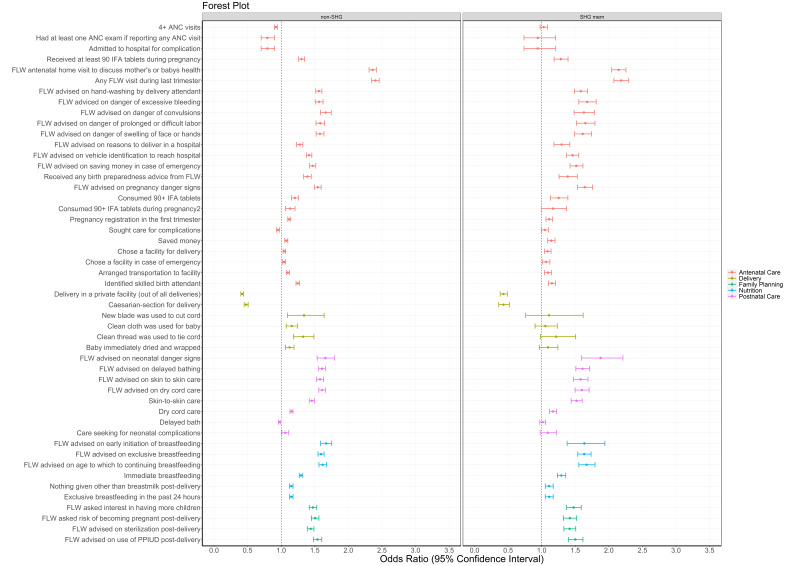
Forest plot of odds ratios and 95% confidence intervals of health, nutrition and sanitation indicators for one increased frontline worker visit stratified by non-self-help group (SHG)-members and SHG members as measured by Community-based Household Surveys rounds 6-9 during scale-up (2014-2017) statewide in Bihar, India. All models presented were adjusted for age of the mother and sex of the focal child. These models also accounted for the study’s complex design by applying study weights. 2 – Out of those who received 90+ IFA during pregnancy. ANC – antenatal care, DPT – diphtheria-pertussis-tetanus, FLW – frontline worker, IFA – iron-folic acid, IPV – inactivated polio vaccine, PPIUD – postpartum intrauterine device.

FLW-beneficiary touchpoints were strong predictors of indicators; 85% of indicators had increased odds in non-members, whereas 72% had increased odds in SHG members. With each additional FLW visit, health indicators rose up to 2-fold. Each indicators’ odds were slightly attenuated in SHG members (**Figure 6**, Table S6 in the **Online Supplementary Document**).

## DISCUSSION

This study in Bihar, India demonstrates that self-reported SHG membership was associated with higher levels of a range of health, nutrition and sanitation-related indicators compared to non-members. The effect sizes were modest (significant ORs ranging from slightly above 1.0 to 1.7) and evident across domains of antenatal care and birth preparedness, postnatal care, complementary feeding/nutrition, sanitation and family planning, but weaker for delivery care and lacking for immunisation. The positive associations are impressive considering the more marginalised status of women in SHGs. According to delivery platform, positive associations were seen most consistently for indicators related to FLW performance, followed by maternal behaviour; only a minority of indicators related to facility care and outreach service delivery improved. This may reflect increased self-efficacy related to SHG membership and enhanced interactions with FLWs but not with health facilities [24,25]. Moreover, there was a dose-dependent increase in benefits with SHG exposure; also, women in SHGs with more children (and potentially more time in SHGs) had better indicators. Conversely younger women had attenuated effects, suggesting the need for focal and age-relevant messaging among younger mothers within SHGs. Lastly, FLW touchpoints were associated with higher levels of most indicators for both SHG members and non-members, but greater increases were seen in non-SHG members, perhaps reflective of the importance of FLW visits, especially in the absence of SHG-related support. Perhaps FLW visits and SHGs serve as supplements for one another, by providing similar types of information, with the potential for increased health benefit through interactions between these platforms. These results largely corroborated earlier findings by Saggurti et al. [24], but furthermore demonstrate effects of SHGs at statewide scale. We found similar increases as Saggurtii et al. in skin-to-skin care, timely initiation of breastfeeding, exclusive breastfeeding, and increased use of modern contraception/family planning, and some differences such as in results on delayed bathing or immunisation uptake. The latter result is perhaps due to differences in the specifics of the measured indicators on similar domains (eg, self-report of 3+ DPT as compared to their other measurements of these same indicators).

Our results also highlight the importance of RMNCHN-related discussions in the SHGs, although cause and effect cannot be established. Women who reported discussing particular topics had higher corresponding reported behaviours. When compared to smaller studies using participatory learning action cycles, our findings underscore the importance of discussions at scale [10,29]. The mechanisms for SHG effects should be explored further. It is not known, for example, whether more mature groups have more positive benefits upon health or whether specific types of groups like those with health-layering may be afforded greater benefit [30]. Future research could also potentially use methods of causal inference to untangle mechanisms related to SHGs.

SHGs may be implemented in different ways to optimise their impact. In the current model, women were primarily older, already having had on average three children. The average age of first birth in rural Bihar is about 16, whereas women in the groups were on average 25. Some group members who are older mothers or even grandmothers could have a positive influence on younger mothers and their health decisions. Our results were similar, although attenuated for younger SHG members, suggesting that this is an area for potential improvement in SHG effectiveness. Given that younger SHG members may be able to change their health behaviours, SHGs could be developed and targeted to younger women in their adolescence, catching women before they have their first child. Recently, JEEViKA adopted a focused initiative to address anemia and family planning in younger women. Perhaps strengthening interventions for younger mothers, including the development of more tailored interventions, messaging and communication strategies, may amplify the impact of SHGs for younger women. Thus, the ability of SHGs to impact RMNCHN outcomes has not yet been maximised. In addition, our analysis does not cover several related key issues, such as diffusion through SHGs, influence of SHGs on service provision and providers at the last mile, optimal group composition and size, cost, proportion of marginalised communities covered, or ratio of group leader to group participants. Our analysis also does not cover several related potential areas of impact such as gender-based violence and other gender equity issues, maternal depression, intra-familial relationships, or women’s social or political empowerment. Further attention to these processes could potentially further increase the effectiveness of the SHG platform, the number of marginalised women served and the geographical area covered.

This evaluation had several strengths. It 1) included a comparison group of non-SHG members for determination of the impact of SHG processes on health, nutrition and sanitation; 2) determined and compared associations between SHG membership and a variety of RMNCHN and sanitation indicators – across the continuum of care from pregnancy to early childhood and through several delivery platforms; and 3) examined the inner working of SHGs, including the impact of specific discussion topics, length of membership, age and parity of group members.

The SHG model of ‘woman-centered’ participatory change at household and community levels is complementary to demand creation through primary health care services which encourage the woman to access services at primary health care centers, often outside her village. Moreover, through the SHG model, the effects of the FLW platform or other systematic primary health care inputs can potentially be magnified, as the knowledge and behaviour change transfer within SHGs is collective instead of only a one-to-one approach. Though demand for increased health care access has only been documented in a minority of SHG evaluations [5,31], and only about one-fifth of indicators of facility care and outreach service delivery were increased in SHG members, there may be a powerful intervention cycle when cyclic gains are borne through greater women’s empowerment, including demand for access to quality services, and strengthening of village-level organisational structures such as the health subcentre platform and health and nutrition sub-committees. This cyclic action derives from the conceptual theories of change that underlie the SHG program model, including participant empowerment wherein SHG members engage in collective action, increasing self-efficacy, encouraging women to have specific dialogues about their circumstances and thereby simultaneously increasing the autonomy of women’s health and linkages of women’s health activities with the provision of services through the primary health care system. While clear conceptually, and although SHGs have been noted to affect health outcomes such as neonatal mortality and maternal mortality [12,32-34], it is unclear whether these groups have activated the primary health care system or whether these improvements occur through other means. A greater question still is the ability of SHGs to sustain their effects at scale. Typically, members of SHGs come from communities with entrenched cultural norms and other barriers; some SHGs in Bihar are focused on marginalised communities such as Scheduled Castes, Scheduled Tribes, and Pasmanda Muslims [35]. Interventions that are demonstrated to work for these groups are especially promising and have a role in improving health equity [36].

Our evaluation also has some limitations. There may be cohort and unmeasured effects that relate to SHG group membership that are the reasons we find the effects seen in this manuscript. Attribution of impact to the program could be clarified if a randomised controlled trial of SHG vs non SHG were conducted. The results of our study show the effect of self-reported participation in SHGs. We lacked data on how many of the women surveyed were offered participation in SHGs; we were only able to measure self-report of inclusion in a SHG (ie, those who agreed to participate). It is possible that the effects of women who chose to enroll and participate in a SHG and reported it to the CHS surveyor may have stronger reported health-related behaviours because they are healthier for unmeasured reasons. While we assessed measures relevant to RMNCHN, the only completely independent data source on the impact of *Ananya* interventions (from Mathematica, see ref [20,37]) was collected for only 2 years from 2012-2014 and was not included here as it predated SHG scale-up. Second, it is not possible to infer causality from a non-randomised study, and unobserved characteristics of SHG members have the potential to confound the association between SHG membership and health indicators. Future randomised, factorial or stepped-wedge designs utilising randomisation could be used to examine health layering upon SHGs and to further tease apart programmatic choices which would lead to greater efficiency of SHGs, such as group size, facilitation including leader qualities, access to care and costs [38]. In addition, we would caution the reader to draw conclusions about attribution based on these data alone. Several co-occurring health and sanitation activities were occurring in Bihar over this time period. For example, the JEEViKA program has conducted several other interventions, such as *Gram Varta*, a participatory-learning-action based model funded by the UK Department for International Development, and *Swabhimaan*, a sanitation initiative funded by UNICEF. In addition, this paper does not specifically present health layering or contrast specific SHG types. We examine specific health-layered interventions upon SHGs in a subsequent paper [15].

## CONCLUSION

This study demonstrates the benefits of SHGs at statewide scale on health, nutrition and sanitation among marginalised women in Bihar, India. These results, coupled with several policy changes and a move towards strengthening the National Rural Health Mission, should be a call to action for the GoB and other state-led government agencies to capitalise on this platform for health, nutrition and sanitation change at scale. Indeed, several drivers of political economy have underscored the role of SHGs and specific political leaders support SHGs; thus, SHGs are growing in Bihar. This work has broad applicability as the SHG platform may potentially be leveraged for a range of RMNCHN and related outcomes.

## Additional material

Online Supplementary Document
