# Supplementary material for: Health impact of self-help groups scaled-up statewide in Bihar, India
Source: J Glob Health. 2020 Dec 19;10(2):021006. doi: 10.7189/jogh.10.021006 (PMC7761401; doi:10.7189/jogh.10.021006)
Supplement: Online Supplementary Document [file jogh-10-021006-s001.pdf]

Health impact of self-help groups scaled-up state-wide in Bihar, India

Supplementary material

Supplemental Figure 1. *Self-help group connections to the primary health care system and frontline workers in India*

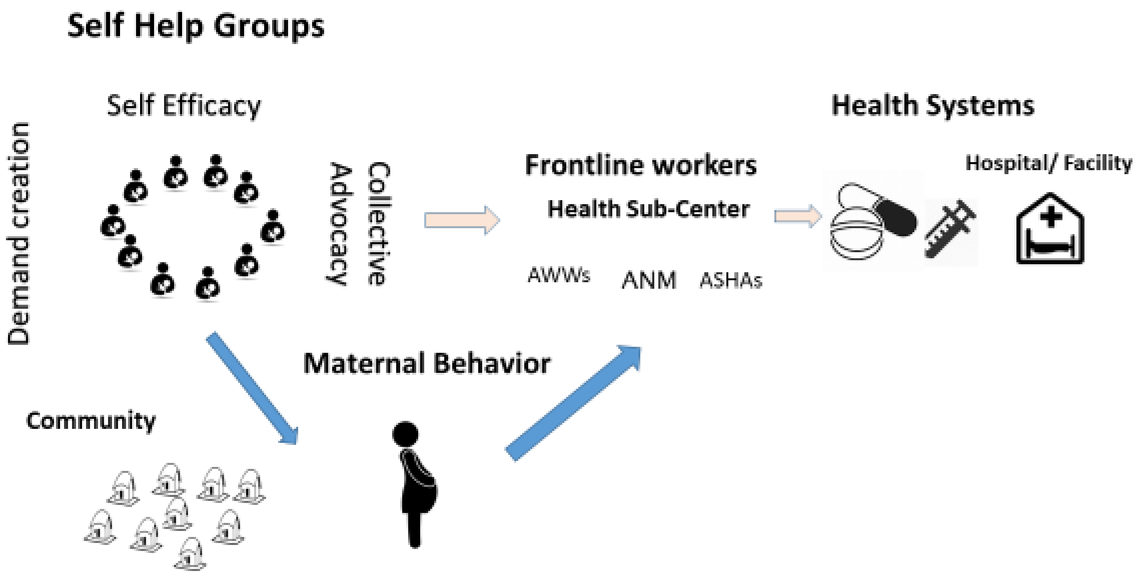

Supplemental Table 1. Health, nutrition and sanitation indicators in the Community-based Household Surveys which were included in SHG analyses

Reproductive, maternal, newborn and child health and nutrition + sanitation indicator	Continuum of care domain	Behaviour Change Pathway	Name	Source (age group, months)
4+ antenatal care (ANC) visits	Antenatal Care	Facility/Outreach Service Delivery	r_fouranc	0-2
Had at least one ANC exam if reporting any ANC visit	Antenatal Care	Facility/Outreach Service Delivery	Anyancexam	0-2
Admitted to hospital for complication	Antenatal Care	Facility/Outreach Service Delivery	admitcompl	0-2
Received at least 90 iron-folic acid (IFA) tablets during pregnancy	Antenatal Care	Facility/Outreach Service Delivery	gotifa90	0-2
Frontline worker (FLW) antenatal home visit to discuss mother's or baby's health	Antenatal Care	Frontline Worker Performance	flwvishlth	0-2
Any FLW visit during last trimester	Antenatal Care	Frontline Worker Performance	any_flw_3rdtrim	0-2
FLW advised on hand-washing by delivery attendant	Antenatal Care	Frontline Worker Performance	advice_hand	0-2
FLW advised on danger of excessive bleeding	Antenatal Care	Frontline Worker Performance	advice_bleed	0-2
FLW advised on danger of convulsions	Antenatal Care	Frontline Worker Performance	advice_conv	0-2
FLW advised on danger of prolonged or difficult labor	Antenatal Care	Frontline Worker Performance	advice_labor	0-2
FLW advised on danger of swelling of face or hands	Antenatal Care	Frontline Worker Performance	advice_edema	0-2
FLW advised on reasons to deliver in a hospital	Antenatal Care	Frontline Worker Performance	advice_facdel	0-2
FLW advised on vehicle identification to reach hospital	Antenatal Care	Frontline Worker Performance	advice_veh	0-2
FLW advised on saving money in case of emergency	Antenatal Care	Frontline Worker Performance	advice_money	0-2
Received any birth preparedness advice from FLW	Antenatal Care	Frontline Worker Performance	anybirthprepadv	0-2

FLW advised on pregnancy danger signs	Antenatal Care	Frontline Worker Performance	advice_preg_signs	0-2
Consumed 90+ IFA tablets	Antenatal Care	Mother's Behaviour	tookifa90	0-2
Consumed 90+ IFA tablets during pregnancy out of those who received 90+ IFA during pregnancy	Antenatal Care	Mother's Behaviour	consifa90	0-2
Pregnancy registration in the first trimester	Antenatal Care	Mother's Behaviour	pregreg1sttrim	0-2
Sought care for complications	Antenatal Care	Mother's Behaviour	r_careseeking_compl	0-2
Saved money	Antenatal Care	Mother's Behaviour	savemoney	0-2
Chose a facility for delivery	Antenatal Care	Mother's Behaviour	pickfac	0-2
Chose a facility in case of emergency	Antenatal Care	Mother's Behaviour	pickemfac	0-2
Arranged transportation to facility	Antenatal Care	Mother's Behaviour	pickvehicle	0-2
Identified skilled birth attendant	Antenatal Care	Mother's Behaviour	picksba	0-2
Delivery in a facility (public or private)	Delivery	Facility/Outreach Service Delivery	pod_facility	0-2
Delivery in a private facility (out of all deliveries)	Delivery	Facility/Outreach Service Delivery	privatefac	0-2
Delivery in a public facility (out of all deliveries)	Delivery	Facility/Outreach Service Delivery	publicfacoffac2	
Caesarian-section for delivery	Delivery	Facility/Outreach Service Delivery	Csection	0-2
New blade was used to cut cord	Delivery	Facility/Outreach Service Delivery	new_blade	0-2
Clean cloth was used for baby	Delivery	Facility/Outreach Service Delivery	clean_cloth	0-2
Clean thread was used to tie cord	Delivery	Facility/Outreach Service Delivery	clean_thread	0-2
Baby weighed at birth	Delivery	Facility/Outreach Service Delivery	r_weighed	0-2
Baby immediately dried and wrapped	Delivery	Mother's Behaviour	dried_wrapped	0-2
Any FLW visits in the first week after delivery	Postnatal Care	Frontline Worker Performance	any_flw_visit_1stweek	0-2
3+ FLW visits in the first week after delivery	Postnatal Care	Frontline Worker Performance	three_flw_visit_1stweek	0-2
FLW advised on neonatal danger signs	Postnatal Care	Frontline Worker Performance	advice_neonatal_signs	0-2
FLW advised on delayed bathing	Postnatal Care	Frontline Worker Performance	advice_delaybath	0-2
FLW advised on skin to skin care	Postnatal Care	Frontline Worker Performance	advice_stsc_new	0-2

FLW advised on dry cord care	Postnatal Care	Frontline Worker Performance	advice_dry_cord	0-2
Skin-to-skin care	Postnatal Care	Mother's Behaviour	stsc_imm_later	0-2
Dry cord care	Postnatal Care	Mother's Behaviour	drycordcare3	0-2
Delayed bath	Postnatal Care	Mother's Behaviour	delay_bath	0-2
Care seeking for neonatal complications	Postnatal Care	Mother's Behaviour	r_careseeking_newborn	0-2
FLW advised on early initiation of breastfeeding	Nutrition	Frontline Worker Performance	advice_bf_pre	0-2
FLW advised on exclusive breastfeeding	Nutrition	Frontline Worker Performance	advice_exc_bf	0-2
FLW advised on age to which to continuing breastfeeding	Nutrition	Frontline Worker Performance	advice_age_bf	0-2
Immediate breastfeeding	Nutrition	Mother's Behaviour	r_bf1	0-2
Nothing given other than breastmilk post-delivery	Nutrition	Mother's Behaviour	exc_breastfd	0-2
Exclusive breastfeeding in the past 24 hours	Nutrition	Mother's Behaviour	EBF_last24hrs	0-2
Initiation of complementary feeding	Nutrition	Mother's Behaviour	initiate_CF	9-11
Age-appropriate initiation of complementary feeding (6-8 months of age)	Nutrition	Mother's Behaviour	age_initiate_cf	9-11
Age-appropriate frequency of complementary feeding 3+ times for 9-11 month-old children	Nutrition	Mother's Behaviour	age_approp_freq_cf	9-11
Fed complementary cereal-based food in past 24 hours	Nutrition	Mother's Behaviour	Cereal	9-11
FLW reminded on vaccine information	Immunization	Frontline Worker Performance	advice_remind_vaccine	0-2
Have immunisation card	Immunization	Facility/Outreach Service Delivery	have_immcard	9-11
Polio (oral polio vaccine3 or inactivated polio vaccine) by card	Immunization	Facility/Outreach Service Delivery	polio3_card	9-11
Diphtheria-pertussis-tetanus3 by card	Immunization	Facility/Outreach Service Delivery	dpt3_card	9-11
FLW asked interest in having more children	Family Planning	Frontline Worker Performance	advice_askfp	0-2
FLW asked risk of becoming pregnant post-delivery	Family Planning	Frontline Worker Performance	advice_pregrisk	0-2

FLW advised on sterilization post-delivery	Family Planning	Frontline Worker Performance	advice_pptl	0-2
FLW advised on use of postpartum intrauterine device post-delivery	Family Planning	Frontline Worker Performance	advice_pptiud	0-2
Modern method of contraception used	Family Planning	Mother's Behaviour	use_modernfp	9-11
Washed hands before feeding child	Sanitation	Mother's Behaviour	Washafterfeed	9-11
Washed hands after using toilet	Sanitation	Mother's Behaviour	Washaftertoil	9-11
Used soap or detergent when washing hands before feed	Sanitation	Mother's Behaviour	WAFuns	9-11
Used soap or detergent when washing hands after toilet	Sanitation	Mother's Behaviour	WATuns	9-11

Supplemental Table 2. Odds ratios and 95% confidence intervals of indicators across the continuum of care and delivery platforms for SHG members compared to non-members as measured by the Community-based Household Surveys rounds 6-9 during scale-up (2014-2017) statewide in Bihar, India					
**RMNCHN + sanitation indicator**	**Continuum of care domain**	**Delivery platform**		**Lower 95% Confidence**	**Upper 95% Confidence**
4+ ANC visits	Antenatal Care	Facility/Outreach Service Delivery	0.842	0.792	0.895
Had at least one ANC exam if reporting any ANC visit	Antenatal Care	Facility/Outreach Service Delivery	0.9	0.649	1.249
Admitted to hospital for complication	Antenatal Care	Facility/Outreach Service Delivery	0.9	0.649	1.249
Received at least 90 IFA tablets during pregnancy	Antenatal Care	Facility/Outreach Service Delivery	0.828	0.75	0.914
FLW antenatal home visit to discuss mother's or baby's health	Antenatal Care	Frontline Worker Performance	1.283	1.221	1.349
Any FLW visit during last trimester	Antenatal Care	Frontline Worker Performance	1.235	1.174	1.299
FLW advised on hand-washing by delivery attendant	Antenatal Care	Frontline Worker Performance	1.324	1.228	1.428
FLW advised on danger of excessive bleeding	Antenatal Care	Frontline Worker Performance	1.348	1.227	1.481
FLW advised on danger of convulsions	Antenatal Care	Frontline Worker Performance	1.379	1.229	1.548
FLW advised on danger of prolonged or difficult labor	Antenatal Care	Frontline Worker Performance	1.3	1.175	1.439
FLW advised on danger of swelling of face or hands	Antenatal Care	Frontline Worker Performance	1.236	1.124	1.359
FLW advised on reasons to deliver in a hospital	Antenatal Care	Frontline Worker Performance	1.317	1.187	1.463
FLW advised on vehicle identification to reach hospital	Antenatal Care	Frontline Worker Performance	1.05	0.972	1.135
FLW advised on saving money in case of emergency	Antenatal Care	Frontline Worker Performance	1.332	1.23	1.442
Received any birth preparedness advice from FLW	Antenatal Care	Frontline Worker Performance	1.271	1.134	1.425
FLW advised on pregnancy danger signs	Antenatal Care	Frontline Worker Performance	1.199	1.103	1.303
Consumed 90+ IFA tablets	Antenatal Care	Mother's Behaviour	0.657	0.576	0.749
Consumed 90+ IFA tablets during pregnancy2	Antenatal Care	Mother's Behaviour	0.897	0.733	1.096
Pregnancy registration in the first trimester	Antenatal Care	Mother's Behaviour	1.292	1.227	1.36
Sought care for complications	Antenatal Care	Mother's Behaviour	0.773	0.729	0.82
Saved money	Antenatal Care	Mother's Behaviour	1.105	1.045	1.169

Chose a facility for delivery	Antenatal Care	Mother's Behaviour	1.218	1.156	1.283
Chose a facility in case of emergency	Antenatal Care	Mother's Behaviour	0.914	0.86	0.973
Arranged transportation to facility	Antenatal Care	Mother's Behaviour	0.977	0.922	1.034
Identified skilled birth attendant	Antenatal Care	Mother's Behaviour	1.197	1.131	1.266
Delivery in a facility (public or private)	Delivery	Facility/Outreach Service Delivery	1	0.945	1.057
Delivery in a private facility (out of all deliveries)	Delivery	Facility/Outreach Service Delivery	0.612	0.567	0.661
Delivery in a public facility (out of all deliveries)	Delivery	Facility/Outreach Service Delivery	1.686	1.558	1.825
Caesarian-section for delivery	Delivery	Facility/Outreach Service Delivery	0.68	0.607	0.762
New blade was used to cut cord	Delivery	Facility/Outreach Service Delivery	1.207	0.77	1.89
Clean cloth was used for baby	Delivery	Facility/Outreach Service Delivery	1.081	0.891	1.311
Clean thread was used to tie cord	Delivery	Facility/Outreach Service Delivery	1.075	0.859	1.345
Baby weighed at birth	Delivery	Facility/Outreach Service Delivery	1.081	1.023	1.142
Baby immediately dried and wrapped	Delivery	Mother's Behaviour	0.949	0.819	1.1
Any FLW visits in the first week after delivery	Postnatal Care	Frontline Worker Performance	1.358	1.291	1.428
3+ FLW visits in the first week after delivery	Postnatal Care	Frontline Worker Performance	1.239	1.151	1.333
FLW advised on neonatal danger signs	Postnatal Care	Frontline Worker Performance	1.311	1.133	1.517
FLW advised on delayed bathing	Postnatal Care	Frontline Worker Performance	1.335	1.235	1.443
FLW advised on skin to skin care	Postnatal Care	Frontline Worker Performance	1.398	1.286	1.521
FLW advised on dry cord care	Postnatal Care	Frontline Worker Performance	1.154	1.065	1.25
Skin-to-skin care	Postnatal Care	Mother's Behaviour	1.378	1.29	1.472
Delayed bath	Postnatal Care	Mother's Behaviour	0.949	0.902	0.998
Care seeking for neonatal complications	Postnatal Care	Mother's Behaviour	0.807	0.711	0.916
FLW advised on early initiation of breastfeeding	Nutrition	Frontline Worker Performance	1.074	0.884	1.304
FLW advised on exclusive breastfeeding	Nutrition	Frontline Worker Performance	1.287	1.197	1.385

FLW advised on age to which to continuing breastfeeding	Nutrition	Frontline Worker Performance	1.187	1.101	1.28
Immediate breastfeeding	Nutrition	Mother's Behaviour	1.461	1.384	1.542
Nothing given other than breastmilk post-delivery	Nutrition	Mother's Behaviour	1.319	1.243	1.398
Exclusive breastfeeding in the past 24 hours	Nutrition	Mother's Behaviour	1.319	1.243	1.398
Initiation of complementary feeding	Nutrition	Mother's Behaviour	0.898	0.852	0.947
Age-appropriate initiation of complementary feeding (6-8 months of age)	Nutrition	Mother's Behaviour	1.059	0.988	1.135
Age-appropriate frequency of complementary feeding3	Nutrition	Mother's Behaviour	1.032	0.98	1.087
Fed complementary cereal-based food in past 24 hours	Nutrition	Mother's Behaviour	0.892	0.845	0.941
FLW reminded on vaccine information	Immunization	Frontline Worker Performance	0.936	0.782	1.119
Have immunisation card	Immunization	Facility/Outreach Service Delivery	0.936	0.782	1.119
Polio (OPV3 or IPV) by card	Immunization	Facility/Outreach Service Delivery	1.091	0.952	1.251
DPT3 by card	Immunization	Facility/Outreach Service Delivery	1.108	0.965	1.273
FLW asked interest in having more children	Family Planning	Frontline Worker Performance	1.399	1.273	1.537
FLW asked risk of becoming pregnant post-delivery	Family Planning	Frontline Worker Performance	1.484	1.357	1.623
FLW advised on sterilization post-delivery	Family Planning	Frontline Worker Performance	1.579	1.455	1.713
FLW advised on use of PPIUD post-delivery	Family Planning	Frontline Worker Performance	1.455	1.323	1.601
Modern method of contraception used	Family Planning	Mother's Behaviour	1.312	1.231	1.399
Washed hands before feeding child	Sanitation	Mother's Behaviour	1.104	1.026	1.187
Washed hands after using toilet	Sanitation	Mother's Behaviour	1.513	1.056	2.167
Used soap or detergent when washing hands before feed	Sanitation	Mother's Behaviour	1.21	1.148	1.276
Used soap or detergent when washing hands after toilet	Sanitation	Mother's Behaviour	1.085	1.031	1.142

Table Legend
All models presented were adjusted for age of the mother and the sex of the focal child. These models also accounted for the study's complex design by applying study weights.
2. out of those who received 90+ IFA during pregnancy
3. 3+ times for 9-11 month-old children

ANC, antenatal care; DPT, diphtheria-pertussis-tetanus; FLW, frontline worker; IFA, iron-folic acid; IPV, inactivated polio vaccine; OPV, oral polio vaccine; PPIUD, postpartum intrauterine device; RMNCHN, reproductive, maternal, newborn and child health and nutrition

*S*upplemental Table 3. Odds ratios and 95% confidence intervals associated with health, nutrition and sanitation indicators for each one-month increase in SHG membership according to the Community-based Household Surveys rounds 6-9, 2014-2017					
**RMNCHN + sanitation indicator**	**Continuum of care Domain**	**Delivery platform**	**Odds ratio associated with 1 month longer SHG membership**	**Lower limit of the 95% confidence ratio**	**Upper limit of the 95% confidence ratio**
4+ ANC visits	Antenatal Care	Facility/Outreach Service Delivery	1.002	1.00	1.006
Had at least one ANC exam if reporting any ANC visit	Antenatal Care	Facility/Outreach Service Delivery	0.994	0.97	1.021
Admitted to hospital for complication	Antenatal Care	Facility/Outreach Service Delivery	0.994	0.97	1.021
Received at least 90 IFA tablets during pregnancy	Antenatal Care	Facility/Outreach Service Delivery	1.004	1.00	1.01
FLW antenatal home visit to discuss mother's or baby's health	Antenatal Care	Frontline Worker Performance	1	1.00	1.003
Any FLW visit during last trimester	Antenatal Care	Frontline Worker Performance	1.001	1.00	1.004
FLW advised on hand-washing by delivery attendant	Antenatal Care	Frontline Worker Performance	1.008	1.00	1.012
FLW advised on danger of excessive bleeding	Antenatal Care	Frontline Worker Performance	1.002	1.00	1.008
FLW advised on danger of convulsions	Antenatal Care	Frontline Worker Performance	1.004	1.00	1.01
FLW advised on danger of prolonged or difficult labor	Antenatal Care	Frontline Worker Performance	1.002	1.00	1.008
FLW advised on danger of swelling of face or hands	Antenatal Care	Frontline Worker Performance	1.006	1.00	1.011
FLW advised on reasons to deliver in a hospital	Antenatal Care	Frontline Worker Performance	1.001	0.99	1.008
FLW advised on vehicle identification to reach hospital	Antenatal Care	Frontline Worker Performance	1.003	1.00	1.008
FLW advised on saving money in case of emergency	Antenatal Care	Frontline Worker Performance	1.006	1.00	1.011
Received any birth preparedness advice from FLW	Antenatal Care	Frontline Worker Performance	1.002	0.99	1.009
FLW advised on pregnancy danger signs	Antenatal Care	Frontline Worker Performance	1.002	1.00	1.008
Consumed 90+ IFA tablets	Antenatal Care	Mother's Behavior	1.001	0.99	1.009

Consumed 90+ IFA tablets during pregnancy2	Antenatal Care	Mother's Behavior	1	0.99	1.013
Pregnancy registration in the first trimester	Antenatal Care	Mother's Behavior	0.998	0.99	1.001
Sought care for complications	Antenatal Care	Mother's Behavior	0.999	1.00	1.003
Saved money	Antenatal Care	Mother's Behavior	1.007	1.00	1.01
Chose a facility for delivery	Antenatal Care	Mother's Behavior	1.002	1.00	1.006
Chose a facility in case of emergency	Antenatal Care	Mother's Behavior	0.996	0.99	1
Arranged transportation to facility	Antenatal Care	Mother's Behavior	1.003	1.00	1.006
Identified skilled birth attendant	Antenatal Care	Mother's Behavior	1.001	1.00	1.005
Delivery in a facility (public or private)	Delivery	Facility/Outreach Service Delivery	1	1.00	1.004
Delivery in a private facility (out of all deliveries)	Delivery	Facility/Outreach Service Delivery	1	0.99	1.005
Caesarian-section for delivery	Delivery	Facility/Outreach Service Delivery	1.004	1.00	1.011
New blade was used to cut cord	Delivery	Facility/Outreach Service Delivery	1.007	0.98	1.033
Clean cloth was used for baby	Delivery	Facility/Outreach Service Delivery	1.01	1.00	1.022
Clean thread was used to tie cord	Delivery	Facility/Outreach Service Delivery	1.018	1.00	1.035
Baby weighed at birth	Delivery	Facility/Outreach Service Delivery	1.005	1.00	1.008
Baby immediately dried and wrapped	Delivery	Mother's Behavior	1.003	0.99	1.013
Any FLW visits in the first week after delivery	Postnatal Care	Frontline Worker Performance	1.002	1.00	1.005
3+ FLW visits in the first week after delivery	Postnatal Care	Frontline Worker Performance	0.999	1.00	1.004
FLW advised on neonatal danger signs	Postnatal Care	Frontline Worker Performance	0.999	0.99	1.008
FLW advised on delayed bathing	Postnatal Care	Frontline Worker Performance	1.007	1.00	1.012
FLW advised on skin to skin care	Postnatal Care	Frontline Worker Performance	1.009	1.00	1.014
FLW advised on dry cord care	Postnatal Care	Frontline Worker Performance	1.006	1.00	1.011
Skin-to-skin care	Postnatal Care	Mother's Behavior	1.003	1.00	1.007
Delayed bath	Postnatal Care	Mother's Behavior	0.996	0.99	1
Care seeking for neonatal complications	Postnatal Care	Mother's Behavior	1.005	1.00	1.014

FLW advised on exclusive breastfeeding	Nutrition	Frontline Worker Performance	1.005	1.00	1.009
FLW advised on age to which to continuing breastfeeding	Nutrition	Frontline Worker Performance	1.001	1.00	1.006
Immediate breastfeeding	Nutrition	Mother's Behavior	1.005	1.00	1.009
Nothing given other than breastmilk post-delivery	Nutrition	Mother's Behavior	1.005	1.00	1.009
Exclusive breastfeeding in the past 24 hours	Nutrition	Mother's Behavior	1.005	1.00	1.009
DPT3 by card	Immunization	Facility/Outreach Service Delivery	1.007	1.00	1.012
FLW asked interest in having more children	Family Planning	Frontline Worker Performance	1.007	1.00	1.012
FLW asked risk of becoming pregnant post-delivery	Family Planning	Frontline Worker Performance	1.004	1.00	1.009
FLW advised on sterilization post-delivery	Family Planning	Frontline Worker Performance	1.006	1.00	1.011
FLW advised on use of PPIUD post-delivery	Family Planning	Frontline Worker Performance	1.003	1.00	1.009

Table Legend

All models presented were adjusted for age of the mother and the sex of the focal child. These models also accounted for the study's complex design by applying study weights.

2. out of those who received 90+ IFA during pregnancy

3. 3+ times for 9-11 month-old children

ANC, antenatal care; DPT, diphtheria-pertussis-tetanus; FLW, frontline worker; IFA, iron-folic acid; PPIUD, postpartum intrauterine device; RMNCHN, reproductive, maternal, newborn and child health and nutrition

Supplemental Table 4. Odds ratios and associated 95% confidence intervals associated with health, nutrition and sanitation indicators for younger SHG members (<25 years old) and older SHG members (≥25 years old) compared to similar aged non-members according to the Community Health Survey, 2014-2017, in Bihar, India

RMNCHN + sanitation indicator	Continuum of care domain	Delivery platform		Lower 95% Confidence	Upper 95% Confidence	Age Category
4+ ANC visits	Antenatal Care	Facility/Outreach Service Delivery	0.866	0.794	0.945	≥25
4+ ANC visits	Antenatal Care	Facility/Outreach Service Delivery	0.823	0.755	0.898	<25
Had at least one ANC exam if reporting any ANC visit	Antenatal Care	Facility/Outreach Service Delivery	1.092	0.705	1.691	≥25
Had at least one ANC exam if reporting any ANC visit	Antenatal Care	Facility/Outreach Service Delivery	0.724	0.437	1.199	<25
Admitted to hospital for complication	Antenatal Care	Facility/Outreach Service Delivery	1.092	0.705	1.691	≥25
Admitted to hospital for complication	Antenatal Care	Facility/Outreach Service Delivery	0.724	0.437	1.199	<25
Received at least 90 IFA tablets during pregnancy	Antenatal Care	Facility/Outreach Service Delivery	0.779	0.678	0.894	≥25
Received at least 90 IFA tablets during pregnancy	Antenatal Care	Facility/Outreach Service Delivery	0.874	0.76	1.005	<25
FLW antenatal home visit to discuss mother's or baby’s health	Antenatal Care	Frontline Worker Performance	1.302	1.217	1.393	≥25
FLW antenatal home visit to discuss mother's or baby’s health	Antenatal Care	Frontline Worker Performance	1.248	1.159	1.344	<25
Any FLW visit during last trimester	Antenatal Care	Frontline Worker Performance	1.229	1.148	1.317	≥25
Any FLW visit during last trimester	Antenatal Care	Frontline Worker Performance	1.227	1.137	1.323	<25
FLW advised on hand-washing by delivery attendant	Antenatal Care	Frontline Worker Performance	1.366	1.234	1.513	≥25
FLW advised on hand-washing by delivery attendant	Antenatal Care	Frontline Worker Performance	1.27	1.136	1.42	<25
FLW adviced on danger of excessive bleeding	Antenatal Care	Frontline Worker Performance	1.351	1.185	1.54	≥25
FLW adviced on danger of excessive bleeding	Antenatal Care	Frontline Worker Performance	1.35	1.18	1.544	<25
FLW advised on danger of convulsions	Antenatal Care	Frontline Worker Performance	1.376	1.172	1.616	≥25
FLW advised on danger of convulsions	Antenatal Care	Frontline Worker Performance	1.38	1.169	1.629	<25
FLW advised on danger of prolonged or difficult labor	Antenatal Care	Frontline Worker Performance	1.303	1.13	1.501	≥25

FLW advised on danger of prolonged or difficult labor	Antenatal Care	Frontline Worker Performance	1.297	1.122	1.499	<25
FLW advised on danger of swelling of face or hands	Antenatal Care	Frontline Worker Performance	1.216	1.066	1.386	≥25
FLW advised on danger of swelling of face or hands	Antenatal Care	Frontline Worker Performance	1.255	1.094	1.44	<25
FLW advised on reasons to deliver in a hospital	Antenatal Care	Frontline Worker Performance	1.302	1.131	1.5	≥25
FLW advised on reasons to deliver in a hospital	Antenatal Care	Frontline Worker Performance	1.332	1.14	1.558	<25
FLW advised on vehicle identification to reach hospital	Antenatal Care	Frontline Worker Performance	1.002	0.901	1.114	≥25
FLW advised on vehicle identification to reach hospital	Antenatal Care	Frontline Worker Performance	1.116	0.997	1.249	<25
FLW advised on saving money in case of emergency	Antenatal Care	Frontline Worker Performance	1.401	1.257	1.562	≥25
FLW advised on saving money in case of emergency	Antenatal Care	Frontline Worker Performance	1.266	1.127	1.423	<25
Received any birth preparedness advice from FLW	Antenatal Care	Frontline Worker Performance	1.219	1.046	1.421	≥25
Received any birth preparedness advice from FLW	Antenatal Care	Frontline Worker Performance	1.332	1.121	1.582	<25
FLW advised on pregnancy danger signs	Antenatal Care	Frontline Worker Performance	1.181	1.053	1.324	≥25
FLW advised on pregnancy danger signs	Antenatal Care	Frontline Worker Performance	1.22	1.08	1.377	<25
Consumed 90+ IFA tablets	Antenatal Care	Mother's Behavior	0.651	0.543	0.78	≥25
Consumed 90+ IFA tablets	Antenatal Care	Mother's Behavior	0.654	0.54	0.791	<25
Consumed 90+ IFA tablets during pregnancy2	Antenatal Care	Mother's Behavior	0.928	0.698	1.233	≥25
Consumed 90+ IFA tablets during pregnancy2	Antenatal Care	Mother's Behavior	0.862	0.648	1.147	<25
Pregnancy registration in the first trimester	Antenatal Care	Mother's Behavior	1.272	1.185	1.366	≥25
Pregnancy registration in the first trimester	Antenatal Care	Mother's Behavior	1.312	1.218	1.413	<25
Sought care for complications	Antenatal Care	Mother's Behavior	0.75	0.693	0.813	≥25
Sought care for complications	Antenatal Care	Mother's Behavior	0.797	0.73	0.869	<25
Saved money	Antenatal Care	Mother's Behavior	1.102	1.022	1.188	≥25

Saved money	Antenatal Care	Mother's Behavior	1.1	1.011	1.196	<25
Chose a facility for delivery	Antenatal Care	Mother's Behavior	1.265	1.178	1.358	≥25
Chose a facility for delivery	Antenatal Care	Mother's Behavior	1.139	1.053	1.231	<25
Chose a facility in case of emergency	Antenatal Care	Mother's Behavior	0.895	0.822	0.974	≥25
Chose a facility in case of emergency	Antenatal Care	Mother's Behavior	0.942	0.861	1.031	<25
Arranged transportation to facility	Antenatal Care	Mother's Behavior	0.95	0.878	1.028	≥25
Arranged transportation to facility	Antenatal Care	Mother's Behavior	0.999	0.918	1.087	<25
Identified skilled birth attendant	Antenatal Care	Mother's Behavior	1.183	1.096	1.277	≥25
Identified skilled birth attendant	Antenatal Care	Mother's Behavior	1.22	1.122	1.326	<25
Delivery in a private facility (out of all deliveries)	Delivery	Facility/Outreach Service Delivery	0.665	0.597	0.74	≥25
Delivery in a private facility (out of all deliveries)	Delivery	Facility/Outreach Service Delivery	0.562	0.503	0.628	<25
Caesarian-section for delivery	Delivery	Facility/Outreach Service Delivery	0.68	0.577	0.802	≥25
Caesarian-section for delivery	Delivery	Facility/Outreach Service Delivery	0.68	0.581	0.796	<25
New blade was used to cut cord	Delivery	Facility/Outreach Service Delivery	1.101	0.624	1.943	≥25
New blade was used to cut cord	Delivery	Facility/Outreach Service Delivery	1.332	0.632	2.805	<25
Clean cloth was used for baby	Delivery	Facility/Outreach Service Delivery	1.116	0.865	1.44	≥25
Clean cloth was used for baby	Delivery	Facility/Outreach Service Delivery	1.005	0.75	1.346	<25
Clean thread was used to tie cord	Delivery	Facility/Outreach Service Delivery	1.026	0.771	1.364	≥25
Clean thread was used to tie cord	Delivery	Facility/Outreach Service Delivery	1.134	0.788	1.632	<25
Baby immediately dried and wrapped	Delivery	Mother's Behavior	0.974	0.796	1.192	≥25
Baby immediately dried and wrapped	Delivery	Mother's Behavior	0.894	0.721	1.109	<25
Any FLW visits in the first week after delivery	Postnatal Care	Frontline Worker Performance	1.365	1.275	1.462	≥25

Any FLW visits in the first week after delivery	Postnatal Care	Frontline Worker Performance	1.342	1.246	1.445	<25
3+ FLW visits in the first week after delivery	Postnatal Care	Frontline Worker Performance	1.303	1.179	1.439	≥25
3+ FLW visits in the first week after delivery	Postnatal Care	Frontline Worker Performance	1.163	1.043	1.296	<25
FLW advised on neonatal danger signs	Postnatal Care	Frontline Worker Performance	1.236	1.015	1.505	≥25
FLW advised on neonatal danger signs	Postnatal Care	Frontline Worker Performance	1.395	1.124	1.732	<25
FLW advised on delayed bathing	Postnatal Care	Frontline Worker Performance	1.425	1.281	1.585	≥25
FLW advised on delayed bathing	Postnatal Care	Frontline Worker Performance	1.237	1.104	1.386	<25
FLW advised on skin to skin care	Postnatal Care	Frontline Worker Performance	1.499	1.335	1.684	≥25
FLW advised on skin to skin care	Postnatal Care	Frontline Worker Performance	1.3	1.15	1.47	<25
FLW advised on dry cord care	Postnatal Care	Frontline Worker Performance	1.178	1.056	1.313	≥25
FLW advised on dry cord care	Postnatal Care	Frontline Worker Performance	1.119	0.994	1.26	<25
Skin-to-skin care	Postnatal Care	Mother's Behavior	1.435	1.309	1.572	≥25
Skin-to-skin care	Postnatal Care	Mother's Behavior	1.32	1.199	1.453	<25
Dry cord care	Postnatal Care	Mother's Behavior	0.991	0.924	1.063	≥25
Dry cord care	Postnatal Care	Mother's Behavior	1.126	1.044	1.214	<25
Delayed bath	Postnatal Care	Mother's Behavior	0.96	0.896	1.028	≥25
Delayed bath	Postnatal Care	Mother's Behavior	0.94	0.872	1.014	<25
Care seeking for neonatal complications	Postnatal Care	Mother's Behavior	0.771	0.646	0.919	≥25
Care seeking for neonatal complications	Postnatal Care	Mother's Behavior	0.842	0.7	1.012	<25
FLW advised on early initiation of breastfeeding	Nutrition	Frontline Worker Performance	1.188	0.923	1.529	≥25
FLW advised on early initiation of breastfeeding	Nutrition	Frontline Worker Performance	0.934	0.688	1.267	<25
FLW advised on exclusive breastfeeding	Nutrition	Frontline Worker Performance	1.295	1.173	1.429	≥25

FLW advised on exclusive breastfeeding	Nutrition	Frontline Worker Performance	1.274	1.142	1.42	<25
FLW advised on age to which to continuing breastfeeding	Nutrition	Frontline Worker Performance	1.159	1.045	1.285	≥25
FLW advised on age to which to continuing breastfeeding	Nutrition	Frontline Worker Performance	1.201	1.075	1.342	<25
Immediate breastfeeding	Nutrition	Mother's Behavior	1.504	1.399	1.617	≥25
Immediate breastfeeding	Nutrition	Mother's Behavior	1.393	1.285	1.511	<25
Nothing given other than breastmilk post-delivery	Nutrition	Mother's Behavior	1.3	1.202	1.407	≥25
Nothing given other than breastmilk post-delivery	Nutrition	Mother's Behavior	1.328	1.216	1.451	<25
Exclusive breastfeeding in the past 24 hours	Nutrition	Mother's Behavior	1.3	1.202	1.407	≥25
Exclusive breastfeeding in the past 24 hours	Nutrition	Mother's Behavior	1.328	1.216	1.451	<25
Initiation of complementary feeding	Nutrition	Mother's Behavior	0.887	0.825	0.953	≥25
Initiation of complementary feeding	Nutrition	Mother's Behavior	0.906	0.837	0.982	<25
Age-appropriate initiation of complementary feeding (6-8 months of age)	Nutrition	Mother's Behavior	1.089	0.992	1.196	≥25
Age-appropriate initiation of complementary feeding (6-8 months of age)	Nutrition	Mother's Behavior	1.018	0.918	1.128	<25
Age-appropriate frequency of complementary feeding3	Nutrition	Mother's Behavior	1.035	0.965	1.111	≥25
Age-appropriate frequency of complementary feeding3	Nutrition	Mother's Behavior	1.031	0.954	1.114	<25
Fed complementary cereal-based food in past 24 hours	Nutrition	Mother's Behavior	0.879	0.818	0.944	≥25
Fed complementary cereal-based food in past 24 hours	Nutrition	Mother's Behavior	0.901	0.831	0.976	<25
FLW reminded on vaccine information	Immunization	Frontline Worker Performance	1.006	0.799	1.268	≥25
FLW reminded on vaccine information	Immunization	Frontline Worker Performance	0.811	0.612	1.074	<25
Have immunisation card	Immunization	Facility/Outreach Service Delivery	1.006	0.799	1.268	≥25
Have immunisation card	Immunization	Facility/Outreach Service Delivery	0.811	0.612	1.074	<25
Polio (OPV3 or IPV) by card	Immunization	Facility/Outreach Service Delivery	1.125	0.944	1.341	≥25

Polio (OPV3 or IPV) by card	Immunization	Facility/Outreach Service Delivery	1.024	0.823	1.272	<25
DPT3 by card	Immunization	Facility/Outreach Service Delivery	1.156	0.968	1.379	≥25
DPT3 by card	Immunization	Facility/Outreach Service Delivery	1.02	0.818	1.271	<25
FLW asked interest in having more children	Family Planning	Frontline Worker Performance	1.301	1.151	1.472	≥25
FLW asked interest in having more children	Family Planning	Frontline Worker Performance	1.52	1.317	1.755	<25
FLW asked risk of becoming pregnant post-delivery	Family Planning	Frontline Worker Performance	1.438	1.279	1.616	≥25
FLW asked risk of becoming pregnant post-delivery	Family Planning	Frontline Worker Performance	1.524	1.329	1.747	<25
FLW advised on sterilization post-delivery	Family Planning	Frontline Worker Performance	1.468	1.324	1.629	≥25
FLW advised on sterilization post-delivery	Family Planning	Frontline Worker Performance	1.695	1.49	1.929	<25
FLW advised on use of PPIUD post-delivery	Family Planning	Frontline Worker Performance	1.423	1.252	1.618	≥25
FLW advised on use of PPIUD post-delivery	Family Planning	Frontline Worker Performance	1.468	1.273	1.694	<25
Modern method of contraception used	Family Planning	Mother's Behavior	1.255	1.162	1.355	≥25
Modern method of contraception used	Family Planning	Mother's Behavior	1.326	1.185	1.483	<25
Washed hands before feeding child	Sanitation	Mother's Behavior	1.136	1.031	1.252	≥25
Washed hands before feeding child	Sanitation	Mother's Behavior	1.058	0.949	1.181	<25
Washed hands after using toilet	Sanitation	Mother's Behavior	1.32	0.81	2.153	≥25
Washed hands after using toilet	Sanitation	Mother's Behavior	1.766	1.034	3.016	<25
Used soap or detergent when washing hands before feed	Sanitation	Mother's Behavior	1.325	1.234	1.424	≥25
Used soap or detergent when washing hands before feed	Sanitation	Mother's Behavior	1.088	1.007	1.176	<25
Used soap or detergent when washing hands after toilet	Sanitation	Mother's Behavior	1.139	1.065	1.219	≥25
Used soap or detergent when washing hands after toilet	Sanitation	Mother's Behavior	1.017	0.94	1.1	<25

Table Legend

All models presented were adjusted for age of the mother and the sex of the focal child. These models also accounted for the study's complex design by applying study weights.
2. out of those who received 90+ IFA during pregnancy
3. 3+ times for 9-11 month-old children
ANC, antenatal care; DPT, diphtheria-pertussis-tetanus; FLW, frontline worker; IFA, iron-folic acid; IPV, inactivated polio vaccine; OPV, oral polio vaccine; PPIUD, postpartum intrauterine device; RMNCHN, reproductive, maternal, newborn and child health and nutrition

Supplemental Table 5. Odds ratios and associated 95% confidence intervals associated health, nutrition and sanitation indicators for SHG members with 0-1 child, 2 children or 3+ children compared to similar aged non-members according to the Community-based Household Surveys, 2014-2017, in Bihar, India

RMNCHN+Sanitation Indicator	Continuum of care domain	Delivery platform	Odds Ratio Estimate	Lower 95% Confidence	Upper 95% Confidence	Number of Children
4+ ANC visits	Antenatal Care	Facility/Outreach Service Delivery	0.93	0.804	1.076	0-1 child
Had at least one ANC exam if reporting any ANC visit	Antenatal Care	Facility/Outreach Service Delivery	1.105	0.531	2.297	0-1 child
Admitted to hospital for complication	Antenatal Care	Facility/Outreach Service Delivery	1.105	0.531	2.297	0-1 child
Received at least 90 IFA tablets during pregnancy	Antenatal Care	Facility/Outreach Service Delivery	0.999	0.789	1.265	0-1 child
FLW antenatal home visit to discuss mother's or baby’s health	Antenatal Care	Frontline Worker Performance	1.282	1.116	1.474	0-1 child
Any FLW visit during last trimester	Antenatal Care	Frontline Worker Performance	1.23	1.066	1.419	0-1 child
FLW advised on hand-washing by delivery attendant	Antenatal Care	Frontline Worker Performance	1.207	0.977	1.492	0-1 child
FLW advised on danger of excessive bleeding	Antenatal Care	Frontline Worker Performance	1.61	1.256	2.063	0-1 child
FLW advised on danger of convulsions	Antenatal Care	Frontline Worker Performance	1.595	1.175	2.165	0-1 child
FLW advised on danger of prolonged or difficult labor	Antenatal Care	Frontline Worker Performance	1.395	1.067	1.824	0-1 child
FLW advised on danger of swelling of face or hands	Antenatal Care	Frontline Worker Performance	1.394	1.082	1.795	0-1 child
FLW advised on reasons to deliver in a hospital	Antenatal Care	Frontline Worker Performance	1.285	0.966	1.709	0-1 child
FLW advised on vehicle identification to reach hospital	Antenatal Care	Frontline Worker Performance	0.999	0.806	1.238	0-1 child
FLW advised on saving money in case of emergency	Antenatal Care	Frontline Worker Performance	1.26	1.011	1.571	0-1 child
Received any birth preparedness advice from FLW	Antenatal Care	Frontline Worker Performance	1.158	0.856	1.566	0-1 child
FLW advised on pregnancy danger signs	Antenatal Care	Frontline Worker Performance	1.348	1.076	1.688	0-1 child
Consumed 90+ IFA tablets	Antenatal Care	Mother's Behavior	0.903	0.665	1.226	0-1 child
Consumed 90+ IFA tablets during pregnancy2	Antenatal Care	Mother's Behavior	1.161	0.705	1.914	0-1 child
Pregnancy registration in the first trimester	Antenatal Care	Mother's Behavior	1.481	1.289	1.702	0-1 child

Sought care for complications	Antenatal Care	Mother's Behavior	0.787	0.667	0.929	0-1 child
Saved money	Antenatal Care	Mother's Behavior	1.123	0.955	1.32	0-1 child
Chose a facility for delivery	Antenatal Care	Mother's Behavior	1.073	0.929	1.239	0-1 child
Chose a facility in case of emergency	Antenatal Care	Mother's Behavior	0.981	0.832	1.157	0-1 child
Arranged transportation to facility	Antenatal Care	Mother's Behavior	0.995	0.847	1.169	0-1 child
Identified skilled birth attendant	Antenatal Care	Mother's Behavior	1.298	1.107	1.522	0-1 child
Delivery in a private facility (out of all deliveries)	Delivery	Facility/Outreach Service Delivery	0.678	0.569	0.808	0-1 child
Caesarian-section for delivery	Delivery	Facility/Outreach Service Delivery	0.812	0.645	1.021	0-1 child
New blade was used to cut cord	Delivery	Facility/Outreach Service Delivery	3.336	0.451	24.651	0-1 child
Clean cloth was used for baby	Delivery	Facility/Outreach Service Delivery	0.991	0.576	1.708	0-1 child
Clean thread was used to tie cord	Delivery	Facility/Outreach Service Delivery	0.928	0.401	2.151	0-1 child
Baby immediately dried and wrapped	Delivery	Mother's Behavior	1.022	0.679	1.536	0-1 child
Any FLW visits in the first week after delivery	Postnatal Care	Frontline Worker Performance	1.286	1.118	1.479	0-1 child
3+ FLW visits in the first week after delivery	Postnatal Care	Frontline Worker Performance	1.132	0.923	1.389	0-1 child
FLW advised on neonatal danger signs	Postnatal Care	Frontline Worker Performance	0.969	0.63	1.49	0-1 child
FLW advised on delayed bathing	Postnatal Care	Frontline Worker Performance	1.239	0.997	1.54	0-1 child
FLW advised on skin to skin care	Postnatal Care	Frontline Worker Performance	1.344	1.064	1.699	0-1 child
FLW advised on dry cord care	Postnatal Care	Frontline Worker Performance	1.165	0.93	1.46	0-1 child
Skin-to-skin care	Postnatal Care	Mother's Behavior	1.284	1.071	1.538	0-1 child
Dry cord care	Postnatal Care	Mother's Behavior	1.053	0.913	1.215	0-1 child
Delayed bath	Postnatal Care	Mother's Behavior	0.883	0.763	1.023	0-1 child
Care seeking for neonatal complications	Postnatal Care	Mother's Behavior	1.021	0.714	1.461	0-1 child
FLW advised on early initiation of breastfeeding	Nutrition	Frontline Worker Performance	1.006	0.615	1.645	0-1 child
FLW advised on exclusive breastfeeding	Nutrition	Frontline Worker Performance	1.228	1.001	1.507	0-1 child
FLW advised on age to which to continuing breastfeeding	Nutrition	Frontline Worker Performance	1.167	0.947	1.439	0-1 child

Immediate breastfeeding	Nutrition	Mother's Behavior	1.289	1.112	1.495	0-1 child
Nothing given other than breastmilk post-delivery	Nutrition	Mother's Behavior	1.359	1.158	1.594	0-1 child
Exclusive breastfeeding in the past 24 hours	Nutrition	Mother's Behavior	1.359	1.158	1.594	0-1 child
Initiation of complementary feeding	Nutrition	Mother's Behavior	0.893	0.777	1.025	0-1 child
Age-appropriate initiation of complementary feeding (6-8 months of age)	Nutrition	Mother's Behavior	1.058	0.894	1.252	0-1 child
Age-appropriate frequency of complementary feeding3	Nutrition	Mother's Behavior	0.989	0.87	1.123	0-1 child
Fed complementary cereal-based food in past 24 hours	Nutrition	Mother's Behavior	0.897	0.78	1.03	0-1 child
Have immunisation card	Immunization	Facility/Outreach Service Delivery	0.63	0.425	0.933	0-1 child
DPT3 by card	Immunization	Facility/Outreach Service Delivery	0.824	0.605	1.124	0-1 child
FLW asked interest in having more children	Family Planning	Frontline Worker Performance	1.418	1.03	1.952	0-1 child
FLW asked risk of becoming pregnant post-delivery	Family Planning	Frontline Worker Performance	1.238	0.908	1.687	0-1 child
FLW advised on sterilization post-delivery	Family Planning	Frontline Worker Performance	1.379	0.986	1.929	0-1 child
FLW advised on use of PPIUD post-delivery	Family Planning	Frontline Worker Performance	0.978	0.702	1.361	0-1 child
Modern method of contraception used	Family Planning	Mother's Behavior	0.731	0.528	1.011	0-1 child
Washed hands before feeding child	Sanitation	Mother's Behavior	1.009	0.841	1.21	0-1 child
Washed hands after using toilet	Sanitation	Mother's Behavior	1.026	0.476	2.213	0-1 child
Used soap or detergent when washing hands before feed	Sanitation	Mother's Behavior	0.981	0.863	1.116	0-1 child
Used soap or detergent when washing hands after toilet	Sanitation	Mother's Behavior	0.954	0.836	1.088	0-1 child
4+ ANC visits	Antenatal Care	Facility/Outreach Service Delivery	1.001	0.888	1.129	2 children
Had at least one ANC exam if reporting any ANC visit	Antenatal Care	Facility/Outreach Service Delivery	0.943	0.468	1.904	2 children
Admitted to hospital for complication	Antenatal Care	Facility/Outreach Service Delivery	0.943	0.468	1.904	2 children
Received at least 90 IFA tablets during pregnancy	Antenatal Care	Facility/Outreach Service Delivery	0.898	0.743	1.085	2 children
FLW antenatal home visit to discuss mother's or baby’s health	Antenatal Care	Frontline Worker Performance	1.168	1.055	1.293	2 children
Any FLW visit during last trimester	Antenatal Care	Frontline Worker Performance	1.146	1.033	1.272	2 children

FLW advised on hand-washing by delivery attendant	Antenatal Care	Frontline Worker Performance	1.249	1.071	1.457	2 children
FLW adviced on danger of excessive bleeding	Antenatal Care	Frontline Worker Performance	1.281	1.059	1.549	2 children
FLW advised on danger of convulsions	Antenatal Care	Frontline Worker Performance	1.344	1.065	1.695	2 children
FLW advised on danger of prolonged or difficult labor	Antenatal Care	Frontline Worker Performance	1.317	1.077	1.611	2 children
FLW advised on danger of swelling of face or hands	Antenatal Care	Frontline Worker Performance	1.19	0.981	1.444	2 children
FLW advised on reasons to deliver in a hospital	Antenatal Care	Frontline Worker Performance	1.166	0.947	1.436	2 children
FLW advised on vehicle identification to reach hospital	Antenatal Care	Frontline Worker Performance	1.213	1.038	1.418	2 children
FLW advised on saving money in case of emergency	Antenatal Care	Frontline Worker Performance	1.307	1.11	1.538	2 children
Received any birth preparedness advice from FLW	Antenatal Care	Frontline Worker Performance	1.202	0.952	1.517	2 children
FLW advised on pregnancy danger signs	Antenatal Care	Frontline Worker Performance	1.188	1.003	1.408	2 children
Consumed 90+ IFA tablets	Antenatal Care	Mother's Behavior	0.552	0.42	0.725	2 children
Consumed 90+ IFA tablets during pregnancy2	Antenatal Care	Mother's Behavior	0.593	0.403	0.872	2 children
Pregnancy registration in the first trimester	Antenatal Care	Mother's Behavior	1.433	1.294	1.587	2 children
Sought care for complications	Antenatal Care	Mother's Behavior	0.831	0.737	0.937	2 children
Saved money	Antenatal Care	Mother's Behavior	1.099	0.98	1.232	2 children
Chose a facility for delivery	Antenatal Care	Mother's Behavior	1.166	1.046	1.299	2 children
Chose a facility in case of emergency	Antenatal Care	Mother's Behavior	1.065	0.944	1.202	2 children
Arranged transportation to facility	Antenatal Care	Mother's Behavior	0.97	0.863	1.089	2 children
Identified skilled birth attendant	Antenatal Care	Mother's Behavior	1.264	1.127	1.417	2 children
Delivery in a private facility (out of all deliveries)	Delivery	Facility/Outreach Service Delivery	0.668	0.575	0.775	2 children
Caesarian-section for delivery	Delivery	Facility/Outreach Service Delivery	0.883	0.724	1.077	2 children
New blade was used to cut cord	Delivery	Facility/Outreach Service Delivery	0.988	0.337	2.899	2 children
Clean cloth was used for baby	Delivery	Facility/Outreach Service Delivery	1.111	0.717	1.72	2 children
Clean thread was used to tie cord	Delivery	Facility/Outreach Service Delivery	1.227	0.705	2.136	2 children
Baby immediately dried and wrapped	Delivery	Mother's Behavior	0.938	0.68	1.296	2 children

Any FLW visits in the first week after delivery	Postnatal Care	Frontline Worker Performance	1.322	1.193	1.464	2 children
3+ FLW visits in the first week after delivery	Postnatal Care	Frontline Worker Performance	1.157	0.993	1.348	2 children
FLW advised on neonatal danger signs	Postnatal Care	Frontline Worker Performance	1.479	1.107	1.975	2 children
FLW advised on delayed bathing	Postnatal Care	Frontline Worker Performance	1.214	1.035	1.423	2 children
FLW advised on skin to skin care	Postnatal Care	Frontline Worker Performance	1.332	1.121	1.582	2 children
FLW advised on dry cord care	Postnatal Care	Frontline Worker Performance	0.996	0.844	1.177	2 children
Skin-to-skin care	Postnatal Care	Mother's Behavior	1.29	1.128	1.476	2 children
Dry cord care	Postnatal Care	Mother's Behavior	1.102	0.994	1.223	2 children
Delayed bath	Postnatal Care	Mother's Behavior	1.032	0.93	1.146	2 children
Care seeking for neonatal complications	Postnatal Care	Mother's Behavior	0.799	0.622	1.024	2 children
FLW advised on early initiation of breastfeeding	Nutrition	Frontline Worker Performance	1.121	0.75	1.675	2 children
FLW advised on exclusive breastfeeding	Nutrition	Frontline Worker Performance	1.238	1.065	1.44	2 children
FLW advised on age to which to continuing breastfeeding	Nutrition	Frontline Worker Performance	1.074	0.921	1.253	2 children
Immediate breastfeeding	Nutrition	Mother's Behavior	1.351	1.209	1.51	2 children
Nothing given other than breastmilk post-delivery	Nutrition	Mother's Behavior	1.146	1.015	1.293	2 children
Exclusive breastfeeding in the past 24 hours	Nutrition	Mother's Behavior	1.146	1.015	1.293	2 children
Initiation of complementary feeding	Nutrition	Mother's Behavior	0.898	0.807	1.001	2 children
Age-appropriate initiation of complementary feeding (6-8 months of age)	Nutrition	Mother's Behavior	1.039	0.903	1.195	2 children
Age-appropriate frequency of complementary feeding3	Nutrition	Mother's Behavior	1.059	0.954	1.174	2 children
Fed complementary cereal-based food in past 24 hours	Nutrition	Mother's Behavior	0.893	0.802	0.995	2 children
Have immunisation card	Immunization	Facility/Outreach Service Delivery	0.8	0.553	1.156	2 children
DPT3 by card	Immunization	Facility/Outreach Service Delivery	1.168	0.874	1.561	2 children
FLW asked interest in having more children	Family Planning	Frontline Worker Performance	1.301	1.063	1.592	2 children
FLW asked risk of becoming pregnant post-delivery	Family Planning	Frontline Worker Performance	1.517	1.255	1.832	2 children

FLW advised on sterilization post-delivery	Family Planning	Frontline Worker Performance	1.378	1.152	1.648	2 children
FLW advised on use of PPIUD post-delivery	Family Planning	Frontline Worker Performance	1.469	1.209	1.785	2 children
Modern method of contraception used	Family Planning	Mother's Behavior	0.941	0.807	1.097	2 children
Washed hands before feeding child	Sanitation	Mother's Behavior	0.994	0.857	1.152	2 children
Washed hands after using toilet	Sanitation	Mother's Behavior	1.64	0.771	3.488	2 children
Used soap or detergent when washing hands before feed	Sanitation	Mother's Behavior	1.142	1.029	1.267	2 children
Used soap or detergent when washing hands after toilet	Sanitation	Mother's Behavior	1.109	0.998	1.232	2 children
4+ ANC visits	Antenatal Care	Facility/Outreach Service Delivery	1.002	0.92	1.092	3+ children
Had at least one ANC exam if reporting any ANC visit	Antenatal Care	Facility/Outreach Service Delivery	0.889	0.574	1.376	3+ children
Admitted to hospital for complication	Antenatal Care	Facility/Outreach Service Delivery	0.889	0.574	1.376	3+ children
Received at least 90 IFA tablets during pregnancy	Antenatal Care	Facility/Outreach Service Delivery	0.847	0.741	0.969	3+ children
FLW antenatal home visit to discuss mother's or baby's health	Antenatal Care	Frontline Worker Performance	1.321	1.239	1.407	3+ children
Any FLW visit during last trimester	Antenatal Care	Frontline Worker Performance	1.262	1.183	1.347	3+ children
FLW advised on hand-washing by delivery attendant	Antenatal Care	Frontline Worker Performance	1.367	1.241	1.505	3+ children
FLW adviced on danger of excessive bleeding	Antenatal Care	Frontline Worker Performance	1.311	1.161	1.481	3+ children
FLW advised on danger of convulsions	Antenatal Care	Frontline Worker Performance	1.334	1.148	1.549	3+ children
FLW advised on danger of prolonged or difficult labor	Antenatal Care	Frontline Worker Performance	1.298	1.136	1.483	3+ children
FLW advised on danger of swelling of face or hands	Antenatal Care	Frontline Worker Performance	1.236	1.093	1.398	3+ children
FLW advised on reasons to deliver in a hospital	Antenatal Care	Frontline Worker Performance	1.394	1.217	1.596	3+ children
FLW advised on vehicle identification to reach hospital	Antenatal Care	Frontline Worker Performance	1.016	0.92	1.122	3+ children
FLW advised on saving money in case of emergency	Antenatal Care	Frontline Worker Performance	1.362	1.23	1.508	3+ children
Received any birth preparedness advice from FLW	Antenatal Care	Frontline Worker Performance	1.331	1.149	1.542	3+ children
FLW advised on pregnancy danger signs	Antenatal Care	Frontline Worker Performance	1.175	1.055	1.308	3+ children

Consumed 90+ IFA tablets	Antenatal Care	Mother's Behavior	0.738	0.619	0.88	3+ children
Consumed 90+ IFA tablets during pregnancy2	Antenatal Care	Mother's Behavior	1.093	0.829	1.442	3+ children
Pregnancy registration in the first trimester	Antenatal Care	Mother's Behavior	1.295	1.211	1.385	3+ children
Sought care for complications	Antenatal Care	Mother's Behavior	0.809	0.75	0.872	3+ children
Saved money	Antenatal Care	Mother's Behavior	1.161	1.082	1.247	3+ children
Chose a facility for delivery	Antenatal Care	Mother's Behavior	1.301	1.217	1.391	3+ children
Chose a facility in case of emergency	Antenatal Care	Mother's Behavior	0.911	0.84	0.988	3+ children
Arranged transportation to facility	Antenatal Care	Mother's Behavior	1.033	0.959	1.113	3+ children
Identified skilled birth attendant	Antenatal Care	Mother's Behavior	1.129	1.05	1.213	3+ children
Delivery in a private facility (out of all deliveries)	Delivery	Facility/Outreach Service Delivery	0.782	0.701	0.871	3+ children
Caesarian-section for delivery	Delivery	Facility/Outreach Service Delivery	0.831	0.693	0.998	3+ children
New blade was used to cut cord	Delivery	Facility/Outreach Service Delivery	1.136	0.675	1.913	3+ children
Clean cloth was used for baby	Delivery	Facility/Outreach Service Delivery	1.079	0.851	1.367	3+ children
Clean thread was used to tie cord	Delivery	Facility/Outreach Service Delivery	1.107	0.856	1.431	3+ children
Baby immediately dried and wrapped	Delivery	Mother's Behavior	0.926	0.77	1.113	3+ children
Any FLW visits in the first week after delivery	Postnatal Care	Frontline Worker Performance	1.371	1.285	1.462	3+ children
3+ FLW visits in the first week after delivery	Postnatal Care	Frontline Worker Performance	1.3	1.184	1.428	3+ children
FLW advised on neonatal danger signs	Postnatal Care	Frontline Worker Performance	1.321	1.096	1.593	3+ children
FLW advised on delayed bathing	Postnatal Care	Frontline Worker Performance	1.39	1.258	1.535	3+ children
FLW advised on skin to skin care	Postnatal Care	Frontline Worker Performance	1.411	1.267	1.573	3+ children
FLW advised on dry cord care	Postnatal Care	Frontline Worker Performance	1.21	1.093	1.34	3+ children
Skin-to-skin care	Postnatal Care	Mother's Behavior	1.445	1.326	1.574	3+ children

Dry cord care	Postnatal Care	Mother's Behavior	1.023	0.958	1.093	3+ children
Delayed bath	Postnatal Care	Mother's Behavior	1.035	0.97	1.103	3+ children
Care seeking for neonatal complications	Postnatal Care	Mother's Behavior	0.821	0.696	0.97	3+ children
FLW advised on early initiation of breastfeeding	Nutrition	Frontline Worker Performance	1.101	0.857	1.413	3+ children
FLW advised on exclusive breastfeeding	Nutrition	Frontline Worker Performance	1.327	1.209	1.457	3+ children
FLW advised on age to which to continuing breastfeeding	Nutrition	Frontline Worker Performance	1.234	1.12	1.36	3+ children
Immediate breastfeeding	Nutrition	Mother's Behavior	1.44	1.344	1.543	3+ children
Nothing given other than breastmilk post-delivery	Nutrition	Mother's Behavior	1.308	1.213	1.41	3+ children
Exclusive breastfeeding in the past 24 hours	Nutrition	Mother's Behavior	1.308	1.213	1.41	3+ children
Initiation of complementary feeding	Nutrition	Mother's Behavior	0.964	0.899	1.034	3+ children
Age-appropriate initiation of complementary feeding (6-8 months of age)	Nutrition	Mother's Behavior	1.109	1.012	1.216	3+ children
Age-appropriate frequency of complementary feeding3	Nutrition	Mother's Behavior	1.085	1.013	1.163	3+ children
Fed complementary cereal-based food in past 24 hours	Nutrition	Mother's Behavior	0.954	0.889	1.023	3+ children
Have immunisation card	Immunization	Facility/Outreach Service Delivery	1.156	0.912	1.463	3+ children
DPT3 by card	Immunization	Facility/Outreach Service Delivery	1.217	1.015	1.46	3+ children
FLW asked interest in having more children	Family Planning	Frontline Worker Performance	1.256	1.121	1.407	3+ children
FLW asked risk of becoming pregnant post-delivery	Family Planning	Frontline Worker Performance	1.329	1.193	1.482	3+ children
FLW advised on sterilization post-delivery	Family Planning	Frontline Worker Performance	1.347	1.223	1.483	3+ children
FLW advised on use of PPIUD post-delivery	Family Planning	Frontline Worker Performance	1.386	1.23	1.56	3+ children
Modern method of contraception used	Family Planning	Mother's Behavior	1.183	1.1	1.273	3+ children
Washed hands before feeding child	Sanitation	Mother's Behavior	1.243	1.131	1.367	3+ children
Washed hands after using toilet	Sanitation	Mother's Behavior	1.687	1.037	2.745	3+ children

Used soap or detergent when washing hands before feed	Sanitation	Mother's Behavior	1.417	1.319	1.522	3+ children
Used soap or detergent when washing hands after toilet	Sanitation	Mother's Behavior	1.205	1.128	1.287	3+ children

Table Legend
All models presented were adjusted for age of the mother and the sex of the focal child. These models also accounted for the study's complex design by applying study weights.
2. out of those who received 90+ IFA during pregnancy
3. 3+ times for 9-11 month-old children
4. 5 models did not parameterize out of 67, 62 reported here
ANC, antenatal care; DPT, diphtheria-pertussis-tetanus; FLW, frontline worker; IFA, iron-folic acid; PPIUD, postpartum intrauterine device; RMNCHN, reproductive, maternal, newborn and child health and nutrition

Supplemental Table 6. Odds ratios and associated 95% confidence intervals associated health, nutrition and sanitation indicators according to FLW visits. Odds displayed are according to 1 increased visit stratified by SHG members and non-members to the Community-based Household Surveys, 2014-2017, in Bihar, India

RMNCHN+Sanitation Indicator	Continuum of care domain	Delivery platform	Odds Ratio Estimate	Lower 95% Confidence	Upper 95% Confidence	SHG membership
4+ ANC visits	Antenatal Care	Facility/Outreach Service Delivery	0.921	0.899	0.942	non-SHG
Had at least one ANC exam if reporting any ANC visit	Antenatal Care	Facility/Outreach Service Delivery	0.795	0.701	0.902	non-SHG
Admitted to hospital for complication	Antenatal Care	Facility/Outreach Service Delivery	0.795	0.701	0.902	non-SHG
Received at least 90 IFA tablets during pregnancy	Antenatal Care	Facility/Outreach Service Delivery	1.303	1.26	1.348	non-SHG
FLW antenatal home visit to discuss mother's or baby’s health	Antenatal Care	Frontline Worker Performance	2.361	2.308	2.416	non-SHG
Any FLW visit during last trimester	Antenatal Care	Frontline Worker Performance	2.402	2.347	2.458	non-SHG
FLW advised on hand-washing by delivery attendant	Antenatal Care	Frontline Worker Performance	1.559	1.514	1.606	non-SHG
FLW advised on danger of excessive bleeding	Antenatal Care	Frontline Worker Performance	1.564	1.507	1.623	non-SHG
FLW advised on danger of convulsions	Antenatal Care	Frontline Worker Performance	1.663	1.585	1.745	non-SHG
FLW advised on danger of prolonged or difficult labor	Antenatal Care	Frontline Worker Performance	1.581	1.518	1.646	non-SHG
FLW advised on danger of swelling of face or hands	Antenatal Care	Frontline Worker Performance	1.577	1.52	1.637	non-SHG
FLW advised on reasons to deliver in a hospital	Antenatal Care	Frontline Worker Performance	1.275	1.229	1.322	non-SHG
FLW advised on vehicle identification to reach hospital	Antenatal Care	Frontline Worker Performance	1.415	1.375	1.456	non-SHG
FLW advised on saving money in case of emergency	Antenatal Care	Frontline worker Performance	1.468	1.423	1.514	non-SHG
Received any birth preparedness advice from FLW	Antenatal Care	Frontline Worker Performance	1.389	1.334	1.447	non-SHG
FLW advised on pregnancy danger signs	Antenatal Care	Frontline Worker Performance	1.545	1.496	1.595	non-SHG
Consumed 90+ IFA tablets	Antenatal Care	Mother's Behavior	1.203	1.152	1.257	non-SHG
Consumed 90+ IFA tablets during pregnancy2	Antenatal Care	Mother's Behavior	1.132	1.062	1.206	non-SHG

Pregnancy registration in the first trimester	Antenatal Care	Mother's Behavior	1.12	1.098	1.142	non-SHG
Sought care for complications	Antenatal Care	Mother's Behavior	0.951	0.929	0.973	non-SHG
Saved money	Antenatal Care	Mother's Behavior	1.075	1.053	1.098	non-SHG
Chose a facility for delivery	Antenatal Care	Mother's Behavior	1.047	1.027	1.067	non-SHG
Chose a facility in case of emergency	Antenatal Care	Mother's Behavior	1.042	1.019	1.066	non-SHG
Arranged transportation to facility	Antenatal Care	Mother's Behavior	1.099	1.075	1.123	non-SHG
Identified skilled birth attendant	Antenatal Care	Mother's Behavior	1.246	1.22	1.273	non-SHG
Delivery in a private facility (out of all deliveries)	Delivery	Facility/Outreach Service Delivery	0.418	0.399	0.438	non-SHG
Caesarian-section for delivery	Delivery	Facility/Outreach Service Delivery	0.477	0.448	0.508	non-SHG
New blade was used to cut cord	Delivery	Facility/Outreach Service Delivery	1.339	1.094	1.639	non-SHG
Clean cloth was used for baby	Delivery	Facility/Outreach Service Delivery	1.156	1.074	1.243	non-SHG
Clean thread was used to tie cord	Delivery	Facility/Outreach Service Delivery	1.327	1.184	1.487	non-SHG
Baby immediately dried and wrapped	Delivery	Mother's Behavior	1.125	1.063	1.191	non-SHG
FLW advised on neonatal danger signs	Postnatal Care	Frontline Worker Performance	1.658	1.535	1.792	non-SHG
FLW advised on delayed bathing	Postnatal Care	Frontline Worker Performance	1.607	1.558	1.657	non-SHG
FLW advised on skin to skin care	Postnatal Care	Frontline Worker Performance	1.578	1.526	1.632	non-SHG
FLW advised on dry cord care	Postnatal Care	Frontline Worker Performance	1.609	1.56	1.66	non-SHG
Skin-to-skin care	Postnatal Care	Mother's Behavior	1.455	1.418	1.493	non-SHG
Dry cord care	Postnatal Care	Mother's Behavior	1.153	1.131	1.175	non-SHG
Delayed bath	Postnatal Care	Mother's Behavior	0.978	0.96	0.997	non-SHG
Care seeking for neonatal complications	Postnatal Care	Mother's Behavior	1.06	1.009	1.112	non-SHG
FLW advised on early initiation of breastfeeding	Nutrition	Frontline Worker Performance	1.667	1.586	1.752	non-SHG
FLW advised on exclusive breastfeeding	Nutrition	Frontline Worker Performance	1.592	1.548	1.638	non-SHG
FLW advised on age to which to continuing breastfeeding	Nutrition	Frontline Worker Performance	1.617	1.562	1.674	non-SHG
Immediate breastfeeding	Nutrition	Mother's Behavior	1.295	1.269	1.322	non-SHG
Nothing given other than breastmilk post-delivery	Nutrition	Mother's Behavior	1.15	1.125	1.176	non-SHG

Exclusive breastfeeding in the past 24 hours	Nutrition	Mother's Behavior	1.15	1.125	1.176	non-SHG
FLW asked interest in having more children	Family Planning	Frontline Worker Performance	1.472	1.417	1.529	non-SHG
FLW asked risk of becoming pregnant post-delivery	Family Planning	Frontline Worker Performance	1.504	1.449	1.561	non-SHG
FLW advised on sterilization post-delivery	Family Planning	Frontline Worker Performance	1.439	1.392	1.487	non-SHG
FLW advised on use of PPIUD post-delivery	Family Planning	Frontline Worker Performance	1.541	1.481	1.604	non-SHG
4+ ANC visits	Antenatal Care	Facility/Outreach Service Delivery	1.03	0.976	1.088	SHG mem
Had at least one ANC exam if reporting any ANC visit	Antenatal Care	Facility/Outreach Service Delivery	0.942	0.736	1.205	SHG mem
Admitted to hospital for complication	Antenatal Care	Facility/Outreach Service Delivery	0.942	0.736	1.205	SHG mem
Received at least 90 IFA tablets during pregnancy	Antenatal Care	Facility/Outreach Service Delivery	1.284	1.185	1.392	SHG mem
FLW antenatal home visit to discuss mother's or baby's health	Antenatal Care	Frontline Worker Performance	2.146	2.042	2.255	SHG mem
Any FLW visit during last trimester	Antenatal Care	Frontline Worker Performance	2.183	2.075	2.296	SHG mem
FLW advised on hand-washing by delivery attendant	Antenatal Care	Frontline Worker Performance	1.582	1.487	1.682	SHG mem
FLW adviced on danger of excessive bleeding	Antenatal Care	Frontline Worker Performance	1.677	1.553	1.811	SHG mem
FLW advised on danger of convulsions	Antenatal Care	Frontline Worker Performance	1.627	1.483	1.786	SHG mem
FLW advised on danger of prolonged or difficult labor	Antenatal Care	Frontline Worker Performance	1.649	1.516	1.793	SHG mem
FLW advised on danger of swelling of face or hands	Antenatal Care	Frontline Worker Performance	1.611	1.491	1.742	SHG mem
FLW advised on reasons to deliver in a hospital	Antenatal Care	Frontline Worker Performance	1.296	1.183	1.418	SHG mem
FLW advised on vehicle identification to reach hospital	Antenatal Care	Frontline Worker Performance	1.459	1.372	1.552	SHG mem
FLW advised on saving money in case of emergency	Antenatal Care	Frontline Worker Performance	1.515	1.422	1.614	SHG mem
Received any birth preparedness advice from FLW	Antenatal Care	Frontline Worker Performance	1.387	1.258	1.529	SHG mem
FLW advised on pregnancy danger signs	Antenatal Care	Frontline Worker Performance	1.641	1.533	1.757	SHG mem
Consumed 90+ IFA tablets	Antenatal Care	Mother's Behavior	1.252	1.129	1.39	SHG mem
Consumed 90+ IFA tablets during pregnancy2	Antenatal Care	Mother's Behavior	1.168	0.997	1.369	SHG mem

Pregnancy registration in the first trimester	Antenatal Care	Mother's Behavior	1.112	1.064	1.161	SHG mem
Sought care for complications	Antenatal Care	Mother's Behavior	1.045	0.994	1.099	SHG mem
Saved money	Antenatal Care	Mother's Behavior	1.142	1.088	1.198	SHG mem
Chose a facility for delivery	Antenatal Care	Mother's Behavior	1.088	1.041	1.139	SHG mem
Chose a facility in case of emergency	Antenatal Care	Mother's Behavior	1.065	1.011	1.122	SHG mem
Arranged transportation to facility	Antenatal Care	Mother's Behavior	1.093	1.041	1.148	SHG mem
Identified skilled birth attendant	Antenatal Care	Mother's Behavior	1.152	1.099	1.207	SHG mem
Delivery in a private facility (out of all deliveries)	Delivery	Facility/Outreach Service Delivery	0.432	0.382	0.488	SHG mem
Caesarian-section for delivery	Delivery	Facility/Outreach Service Delivery	0.432	0.36	0.519	SHG mem
New blade was used to cut cord	Delivery	Facility/Outreach Service Delivery	1.109	0.761	1.617	SHG mem
Clean cloth was used for baby	Delivery	Facility/Outreach Service Delivery	1.054	0.902	1.232	SHG mem
Clean thread was used to tie cord	Delivery	Facility/Outreach Service Delivery	1.216	0.981	1.506	SHG mem
Baby immediately dried and wrapped	Delivery	Mother's Behavior	1.093	0.964	1.241	SHG mem
FLW advised on neonatal danger signs	Postnatal Care	Frontline Worker Performance	1.876	1.593	2.209	SHG mem
FLW advised on delayed bathing	Postnatal Care	Frontline Worker Performance	1.608	1.509	1.714	SHG mem
FLW advised on skin to skin care	Postnatal Care	Frontline Worker Performance	1.578	1.474	1.689	SHG mem
FLW advised on dry cord care	Postnatal Care	Frontline Worker Performance	1.598	1.497	1.706	SHG mem
Skin-to-skin care	Postnatal Care	Mother's Behavior	1.518	1.438	1.603	SHG mem
Dry cord care	Postnatal Care	Mother's Behavior	1.167	1.117	1.219	SHG mem
Delayed bath	Postnatal Care	Mother's Behavior	1.014	0.972	1.059	SHG mem
Care seeking for neonatal complications	Postnatal Care	Mother's Behavior	1.091	0.977	1.218	SHG mem
FLW advised on early initiation of breastfeeding	Nutrition	Frontline Worker Performance	1.635	1.378	1.941	SHG mem
FLW advised on exclusive breastfeeding	Nutrition	Frontline Worker Performance	1.633	1.537	1.735	SHG mem
FLW advised on age to which to continuing breastfeeding	Nutrition	Frontline Worker Performance	1.669	1.553	1.794	SHG mem
Immediate breastfeeding	Nutrition	Mother's Behavior	1.293	1.232	1.358	SHG mem
Nothing given other than breastmilk post-delivery	Nutrition	Mother's Behavior	1.11	1.053	1.171	SHG mem

Exclusive breastfeeding in the past 24 hours	Nutrition	Mother's Behavior	1.11	1.053	1.171	SHG mem
FLW asked interest in having more children	Family Planning	Frontline Worker Performance	1.476	1.372	1.589	SHG mem
FLW asked risk of becoming pregnant post-delivery	Family Planning	Frontline Worker Performance	1.418	1.324	1.519	SHG mem
FLW advised on sterilization post-delivery	Family Planning	Frontline Worker Performance	1.415	1.33	1.507	SHG mem
FLW advised on use of PPIUD post-delivery	Family Planning	Frontline Worker Performance	1.501	1.396	1.614	SHG mem

Table Legend
All models presented were adjusted for age of the mother and the sex of the focal child. These models also accounted for the study's complex design by applying study weights.
2. out of those who received 90+ IFA during pregnancy
3. 3+ times for 9-11 month-old children
ANC, antenatal care; DPT, diphtheria-pertussis-tetanus; FLW, frontline worker; IFA, iron-folic acid; PPIUD, postpartum intrauterine device; reproductive, maternal, newborn and child health and nutrition, RMNCHN
